# Genome-Wide Analysis of the bZIP Gene Family Identifies Two ABI5-Like bZIP Transcription Factors, BrABI5a and BrABI5b, as Positive Modulators of ABA Signalling in Chinese Cabbage

**DOI:** 10.1371/journal.pone.0158966

**Published:** 2016-07-14

**Authors:** Yili Bai, Wenbo Zhu, Xiaochen Hu, Congcong Sun, Yanlin Li, Dandan Wang, Qinhu Wang, Guoliang Pei, Yanfeng Zhang, Aiguang Guo, Huixian Zhao, Haibin Lu, Xiaoqian Mu, Jingjiang Hu, Xiaona Zhou, Chang Gen Xie

**Affiliations:** 1 State Key Laboratory of Crop Stress Biology for Arid Areas, College of Life Science, Northwest A&F University, Yangling, Shaanxi, 712100, China; 2 Hybrid Rapeseed Research Centre of Shaanxi Province, Yangling, Shaanxi, 712100, China; 3 College of Agronomy, Northwest A&F University, Yangling, Shaanxi, 712100, China; 4 Department of Horticulture and Landscape Architecture, Purdue University, West Lafayette, IN, 47907, United States of America; National Taiwan University, TAIWAN

## Abstract

bZIP (basic leucine zipper) transcription factors coordinate plant growth and development and control responses to environmental stimuli. The genome of Chinese cabbage (*Brassica rapa*) encodes 136 putative bZIP transcription factors. The bZIP transcription factors in *Brassica rapa* (BrbZIP) are classified into 10 subfamilies. Phylogenetic relationship analysis reveals that subfamily A consists of 23 BrbZIPs. Two BrbZIPs within subfamily A, Bra005287 and Bra017251, display high similarity to ABI5 (ABA Insensitive 5). Expression of subfamily A BrbZIPs, like *BrABI5a* (Bra005287/BrbZIP14) and *BrABI5b* (Bra017251/BrbZIP13), are significantly induced by the plant hormone ABA. Subcellular localization assay reveal that both BrABI5a and BrABI5b have a nuclear localization. BrABI5a and BrABI5b could directly stimulate ABA Responsive Element-driven *HIS* (a *HIS3* reporter gene, which confers His prototrophy) or LUC (*LUCIFERASE*) expression in yeast and Arabidopsis protoplast. Deletion of the bZIP motif abolished BrABI5a and BrABI5b transcriptional activity. The ABA insensitive phenotype of Arabidopsis *abi5-1* is completely suppressed in transgenic lines expressing BrABI5a or BrABI5b. Overall, these results suggest that ABI5 orthologs, BrABI5a and BrABI5b, have key roles in ABA signalling in Chinese cabbage.

## Introduction

Cooperation between transcription factors and the core transcription enzyme, RNA polymerase, initiates gene expression in eukaryote organisms. Transcription factors comprise approximately 3.5–7.0% of the genome [1]. The plant genome has a number of transcription factors families, such as MYB, AP2, bHLH, WRKY, NAC, and MADS [2]. The bZIP (Basic Leucine Zipper) family is one of the largest and most diverse transcription factor families in plants [3,4]. All bZIP family members share a highly conserved domain, which contains a basic region and a Leu zipper, known as the bZIP domain. The basic region contains a N-x7-R/K-x9 motif that directly binds to DNA and determines its nuclear localization. The leucine zipper forms an amphipathic surface, which plays an important role in bZIP transcription factors dimerization [3,4,5]. In addition, bZIP transcription factors have other conserved motifs besides the bZIP domain to modulate their transcriptional activity [3,5]. For example, phosphorylation of a conserved Ser or Thr in the R-X-X-S/T motif in many subfamily A bZIP factors activates target gene expression [6,7,8,9].

In Arabidopsis, 13 bZIP factors are divided into subfamily A, which includes ABI5 (ABA Insensitive 5) and ABFs (Abcisic Acid Responsive Element Binding Factors), also known as AREBs (ABA-Responsive Element Binding Proteins) [3]. ABI5 and ABFs have crucial roles to activate plant ABA (Abcisic Acid) signalling [10,11,12,13,14,15,16,17,18]. In addition, post-translational modifications fine-tune ABI5- and ABF-like bZIP transcription factors signalling through cellular processes, such as phosphorylation [6,7,8,14,19,20,21], ubiquitination-mediated protein stability [19,22,23,24], sumolation [25], and S-nitrosylation [26]. In the past decade, a number of ABI5- or ABF-like bZIP transcription factors in other plant species have been characterized, such as HvABI5 from barley [27] and OsABF1, OsABF2, and OsABI5 from rice [28,29,30,31,32], VvABF2 from grape (*Vitis vinifera*) [33], and BolABI5 from cabbage (*Brassica oleracea*)[9]. As expected, many ABI5- or ABF-like bZIP transcription factors demonstrate a pivotal role in ABA responses [9,27,28,29,30,31,32,33].

A number of bZIP factors have been identified in plant genomes, such as 75 bZIP genes in Arabidopsis [3], 89 in rice [4], 88 in Sorghum [1],125 in maize [34], 100 in castor bean [35], 64 in cucumber [36], 55 in grapevine [5], 89 in Barley [37] and 96 in grass (*Brachypodium distachyon*) [38]. Chinese cabbage (*Brassica rapa*) is a dominant vegetable crop consumed in northern China during winter [39,40]. Recently, a total of 136 bZIP factors have been annotated in *Brassica rapa* [41].

So far, few bZIPs have been characterized in *Brassica rapa* [41,42,43] and its relatives, *Brassica oleracea* [9], *Brassica napus* (oil rape) [44,45,46] and *Brassica juncea* [47]. Moreover, it is still unknown whether the subfamily A BrbZIPs (the bZIP factors in *Brassica rapa*) modulate ABA responses. To understand the evolutionary relationship among bZIP transcription factors in *Brassica rapa*, we constructed a phylogenetic tree and classified them [1,3,4,5,35,36,37,38]. In addition, we characterized two ABI5-like BrbZIPs, *BrABI5a* (Bra005287/BrbZIP14) and *BrABI5b* (Bra017251/BrbZIP13).

## Materials and Methods

### Protein Properties and Phylogenetic Analysis

To verify the bZIP domain in putative BrbZIPs[41], online tools such as to search for conserved domains within a protein or coding nucleotide sequence (http://www.ncbi.nlm.nih.gov/Structure/cdd/wrpsb.cgi) [48], SMART (Simple Modular Architecture Research Tool, http://smart.embl-heidelberg.de/), Pfam (http://pfam.xfam.org/) and HMMER (Profile hidden Markov models for biological sequence analysis, http://www.ebi.ac.uk/Tools/hmmer/) were used to perform bZIP domain predictions. Proteins which showed the presence of bZIP domain with confidence (*E*-value <1.0) were selected for further analysis.

The molecular weight (kDa) and isoelectric point (pI) of BrbZIPs were calculated by DNAstar. The conserved motifs and protein architecture were predicted by the MEME (Multiple Em for Motif Elicitation) tool (http://meme-suite.org/tools/meme) with parameters set: optimum motif width ≥6 and ≤200, maximum number of motifs 25 as previously described [35]. All AtbZIPs and BrbZIPs were aligned with the MUSCLE tool and the maximum likelihood trees were generated using MEGA 5.0 as previously described [9,35,49,50].

### Chromosome Location and Intron/Exon Organization of *BrbZIP* Genes

The physical positional information of each *BrbZIP*s genes was downloaded from the *Brassica rapa* database (BRAD, http://brassicadb.org/brad/index.php). Locations of *BrbZIP* genes on *Brassica rapa* chromosomes were then deciphered with MapChart 2.2 tool (http://www.wageningenur.nl/en/show/Mapchart.htm)[51].

The CDSs (coding sequences) of the *BrbZIP* genes were also downloaded from *Brassica rapa* database (BRAD, http://brassicadb.org/brad/index.php). The CDSs of the *BrbZIP* genes were used as the queries for local BLAST to search against the whole genome assembly of *Brassica rapa* (*B*.*rapa*_Chromosome_V1.5). The genomic sequences of *BrbZIP* genes were then retrieved. The online Gene Structure Display Server (GSDS2.0, http://gsds.cbi.pku.edu.cn/) [52] was used to decipher the architectures of *BrbZIP* genes.

### qRT-PCR (Real-Time Quantitative RT-PCR) Analysis

Total RNA was extracted from samples after treatments with TRIzol reagent (TaKaRa). Total RNA was then treated with RNase-free DNase (TaKaRa) to remove DNA, and used for reverse transcription with PrimeScript™ RT Master Mix (Perfect Real Time, TaKaRa). Then, real-time qRT-PCR was performed using a CFX96 real-time PCR machine (Bio-Rad, Hercules, CA, USA) and SYBR Premix Ex Taq kit (TaKaRa) to monitor double-stranded DNA products as previously described [8,9,49,50]. Data from real-time PCR was analyzed by the software (Bio-Rad CFX Manager) and the standard curve method (delta‐delta ct value) was used for calculating the relative expression of experimental genes normalized to the expression of cabbage *ACTIN2* (*BrACTIN2*/Bra037560) according to the manufacturer’s instructions. The primers used for qRT-PCR are listed online in S1 Table.

### Plasmid Construction

To construct Myc-tagged BrABI5a and Myc-tagged BrABI5b, full-length CDSs were amplified via RT-PCR at first. The sequence-confirmed, full-length CDSs of *BrABI5a* and *BrABI5b* were then cloned into *Bam*HⅠ and *Sal*Ⅰ sites of the binary vector p1307-6Myc as previously described [8,9,49,50].

To make GFP-tagged BrABI5a and BrABI5b (BrABI5a-GFP and BrABI5b-GFP), the CDSs were removed from p1307-6Myc-BrABI5a and p1307-6Myc-BrABI5b and then inserted into the Cam-35S-GFP vector between the *Bam*HⅠ and *Sal*Ⅰsites, resulting in a C-terminal fusion to GFP.

To express BrABI5a and BrABI5b in yeast, the CDSs of *BrABI5a* and *BrABI5b* were cloned into the pPC86 vector between *Sal*Ⅰ and *Eco*RⅠ sites as previously described [8,9]. To delete the bZIP motif on BrABI5a and BrABI5b, we truncated BrABI5a (1–352 aa, BrABI5aΔbZIP) and BrABI5b (1–310 aa, BrABI5bΔbZIP) via PCR amplification. The products *BrABI5aΔbZIP* and *BrABI5bΔbZIP* were also inserted into the pPC86 vector between *Sal*Ⅰ and *Eco*RⅠ sites respectively. The primers used to construct the plasmids are listed online in S2 Table. All plasmids were confirmed by sequencing to avoid cloning errors.

### Subcellular Localization

The Cam-35S-BrABI5a-GFP and Cam-35S-BrABI5b-GFP vectors were introduced into the *Agrobacterium tumefaciens* strain GV3101 and then infiltrated into 5- to 6-week-old *Nicotiana benthamiana* leaves for transient expression as previously described [9,53]. The *Agrobacterium* strains were infiltrated at an OD_600_ of 0.5. For microscopic analyses, leaf discs were cut 3 days after infiltration. Cells from the lower epidermis were analyzed at room temperature with 20% glycine as the imaging medium. GFP-fluorescence signals were examined under an inverted Zeiss LSM 510 META fluorescence confocal microscope.

### Yeast One-Hybrid Assay

The yeast strain yWAM2 was used to perform a yeast one-hybrid assay. Yeast transformation and growth assays were performed according to the Yeast Protocols Handbook provided by Clontech. Briefly, pPC86-BrABI5a or pPC86-BrABI5b or pPC86-BrABI5aΔbZIP or pPC86-BrABI5bΔbZIP combined with either pRS315-6×ABRE-HIS or pRS315-HIS were transformed into the yeast strain yWAM2 with the lithium acetate/single-stranded carrier DNA/polyethylene glycol method. The transformed yeast cells were selected on synthetic complete medium lacking leucine and tryptophan (SC-LW). DNA binding and transactivation were determined by measuring the growth of serial dilutions of transformed yeast cells on synthetic complete medium lacking leucine, tryptophan and histidine (SC-LWH) for 2–3 days. All of these were performed as previously described [8,9].

### Transactivation Activity of BrABI5a and BrABI5b *In Vivo*

To detect the transactivation activity of BrABI5a and BrABI5b *in planta*, the p*EM6*-fLUC reporter system was recruited as previously described [8]. The p1307-6Myc-BrABI5a, p1307-6Myc-BrABI5b, p1307-6Myc-BrABI5aΔbZIP and p1307-6Myc-BrABI5bΔbZIP constructs were used as effector plasmids. The reference plasmid 35S-rLUC was obtained from Promega. Combinations of purified plasmids (via a Plasmid Maxiprep Kit, Vigorous Biotechnology) were introduced into Arabidopsis leaf mesophyll protoplasts according to the PEG-Ca^2+^ protocol. Transfected cells were then cultured for 12 to 16 h in the absence or presence of 5 μM ABA. Relative LUC activity was determined according to a Dual-Luciferase Reporter Assay Protocol provided by Promega. The usage amount of combinations of purified plasmids is as follows: *pEM6*-fLUC (7 μg per transfection), 35S-rLUC (2μg per transfection), effector plasmids of BrABI5a and BrABI5b (p1307-6Myc-BrABI5a, p1307-6Myc-BrABI5b, p1307-6Myc-BrABI5aΔbZIP or p1307-6Myc-BrABI5bΔbZIP, 3μg per transfection). The entire transactivation assay *in planta* was performed as previously described [8].

### Construction of BrABI5a and BrABI5b Transgenic Plants

The p1307-6Myc-BrABI5a and p1307-6Myc-BrABI5b constructs were transformed into the *Agrobacterium tumefaciens* strain GV3101 and then infiltrated into *abi5-1* plants with the floral dip method. Seeds (T_0_) from infiltrated plants were selected on MS medium containing 25 μg/L hygromycin (Roche). Homozygous T_3_ plants (derived from different T_1_ transformants) of *abi5-1* harboring each construct were used for ABA inhibition of seed germination as previously described [8,9].

### Stress Treatments and ABA Inhibition of Seed Germination

For abiotic stresses and exogenous ABA treatments, 11-day-old seedlings of Chinese cabbage (Chiifu-401-42, obtained from Hybrid Rapeseed Research Centre of Shaanxi Province, Yangling, Shaanxi, China) were treated with abiotic stresses (300 mM NaCl, -1.7 MPa PEG-8000) and hormones (0.1 mm ABA), followed by sampling at 0, 4, 8,12,16,20 and 24 hr respectively. Drought treatment was achieved by leaving the intact seedlings in the air without supplemented with water, followed by sampling at 0, 4, 8, 12, 16, 20 and 24 hr.

ABA inhibition of seed germination was performed as previously described [8,9,13,30,54]. Briefly, seeds of Wassilewskija (Ws-2), *abi5-1* and transgenic plants harbouring 6*Myc*-*BrABI5a* or 6*Myc*-*BrABI5b* (*abi5-1*:: 6*Myc*-*BrABI5a* or *abi5-1*:: 6*Myc*-*BrABI5b*) were sterilized in a solution containing 20% sodium hypochlorite and 0.1% Triton X-100 for 10 min, washed five times with sterile water, and sown on MS medium (Phytotech) with 0.3% Phytagel (Sigma-Aldrich) with different concentrations of ABA (Sigma-Aldrich). The plates were incubated in growth chambers at 4°C for 4 days followed by incubation at 23°C under continual illumination. To quantify the percentage of seedlings with green cotyledons, seeds were sown on MS medium containing different concentrations of ABA and analyzed on the indicated days after stratification. For radicle emergence assays, seeds were sown on MS medium without sucrose and determined 3 days after stratification.

### Western Blot Analysis

The total protein of transgenic seedlings harbouring 6*Myc*-*BrABI5a* or 6*Myc*-*BrABI5b* (*abi5-1*:: 6*Myc*-*BrABI5a* or *abi5-1*:: 6*Myc*-*BrABI5b*) was homogenized in IP buffer (10 mM Tris-HCl, pH 7.5, 0.5% Nonidet-P40, 2 mM EDTA, 150 mM NaCl, 1× protease inhibitor cocktail (Roche)) and cleared by centrifugation at 13,000 rpm for 10 min at 4°C. Twenty micrograms of the resulting soluble protein was then separated by 12% SDS-PAGE and blotted onto a polyvinylidene difluoride membrane (Millipore). The blot was immediately blocked in 5% non-fat milk for 1 hr. After washing three times with PBST buffer, the blot was probed with anti-Myc antibody (Abmart, 1:5,000 dilutions) and then horseradish peroxidase-conjugated anti-mouse antiserum (Abmart, 1:5,000 dilutions) as a secondary antibody. Signals of horseradish peroxidase-conjugated anti-mouse antiserum with ECL^TM^ chemiluminescence substrate (Abmart) were detected by film as previously described [9].

## Results

### Phylogenetic Analysis of the bZIP Transcription Factor Family in Chinese Cabbage

**Fig 1:**
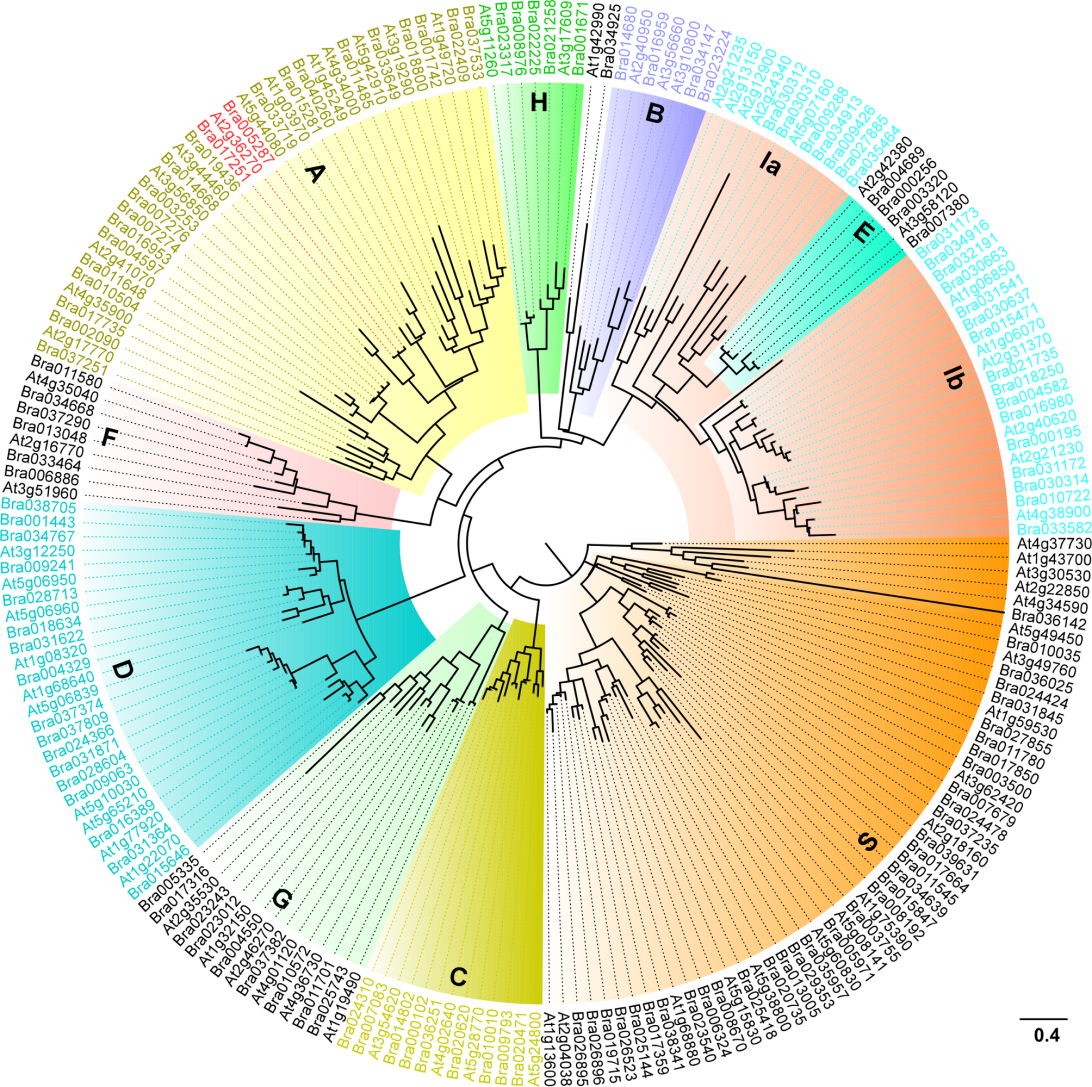
The phylogenetic tree of bZIP transcription factors between Chinese cabbage (*Brassica rapa*) and Arabidopsis. The 136 BrbZIPs and 75 AtbZIPs protein sequences were aligned by the MUSCLE tool; and the maximum likelihood tree was generated using MEGA 5.0. The 10 distinct subfamilies were designated as A~S and labeled with different colored branches respectively.

To gain evolutionary insight into the phylogenetic relationship in bZIP transcription factors between *Brassica rapa* and *Arabidopsis thaliana*, we constructed a phylogenetic tree with 136 BrbZIP and 75 AtbZIP transcription factors (Fig 1) and classified them using a previously reported method [1,3,4,5,35,36,37,38]. Phylogenetic analysis indicated that 23 BrbZIPs were categorized into subfamily A (Fig 1 and S3 Table), 4 proteins in BrbZIPs were closely related to subfamily B, 9 closely related to subfamily C, 17 in subfamily D, 4 in subfamily E, 6 in subfamily F, 9 in subfamily G, 5 in subfamily H, 23 in subfamily I, and 35 in subfamily S (Fig 1 and S3 Table).

### Chromosomal Location of *BrbZIP* Genes in Chinese Cabbage

To determine the chromosomal distribution of *BrbZIP* genes, we used MapChart 2.2. The identified 136 *BrbZIP* genes, except Bra040260/BrbZIP17, were mapped on the A01 to A10 chromosomes of *Brassica rapa* (S1 Fig). Bra040260/BrbZIP17 could not be located on any chromosome of *Brassica rapa*, though it was anchored on Scaffold00019. *BrbZIP* genes are scattered on each chromosome in *Brassica rapa*, but their distribution density differs. The 15.5–25.1 Mb region of A06, 0.3–5.5 Mb region of A09 and 26.1–37.7 Mb region of A09 expressed a higher density of *BrbZIP* genes. There was at least one *BrbZIP* cluster on each chromosome, although A03 had 3 *BrbZIP* clusters (S1 Fig). Interestingly, two pairs of *BrbZIP* genes occurred in tandem on chromosome A09. One pair of tandem duplicated BrbZIPs, Bra026895/BrbZIP135 and Bra026896/BrbZIP134, was categorized as putative subfamily I BrbZIPs members (S3 Table) and showed 100% identity with each other at the cDNA and genomic DNA sequence level. The other pair, Bra007274/BrbZIP8 and Bra007276/BrbZIP9, comprised two putative subfamily A BrbZIPs (S3 Table) that displayed 98.23% (match/nonmatch = 834/15) and 98.79% (match/nonmatch = 1222/15) identity with each other at the cDNA and genomic DNA sequence level, respectively.

We further analyzed the chromosomal distributions of subfamily A *BrbZIP*s. As a result, 3 genes of subfamily A *BrbZIPs* mapped on A01, 2 on A03, 3 on A04, 3 on A05, 4 on A06, 2 on A07, 1 on A08, 3 on A09, and 1 on A10 (S1 Fig, green and red marked genes). Surprisingly, no putative subfamily A *BrbZIP* genes were located on A02.

### Gene Structure Analysis of Subfamily A *BrbZIP* Genes

**Fig 2:**
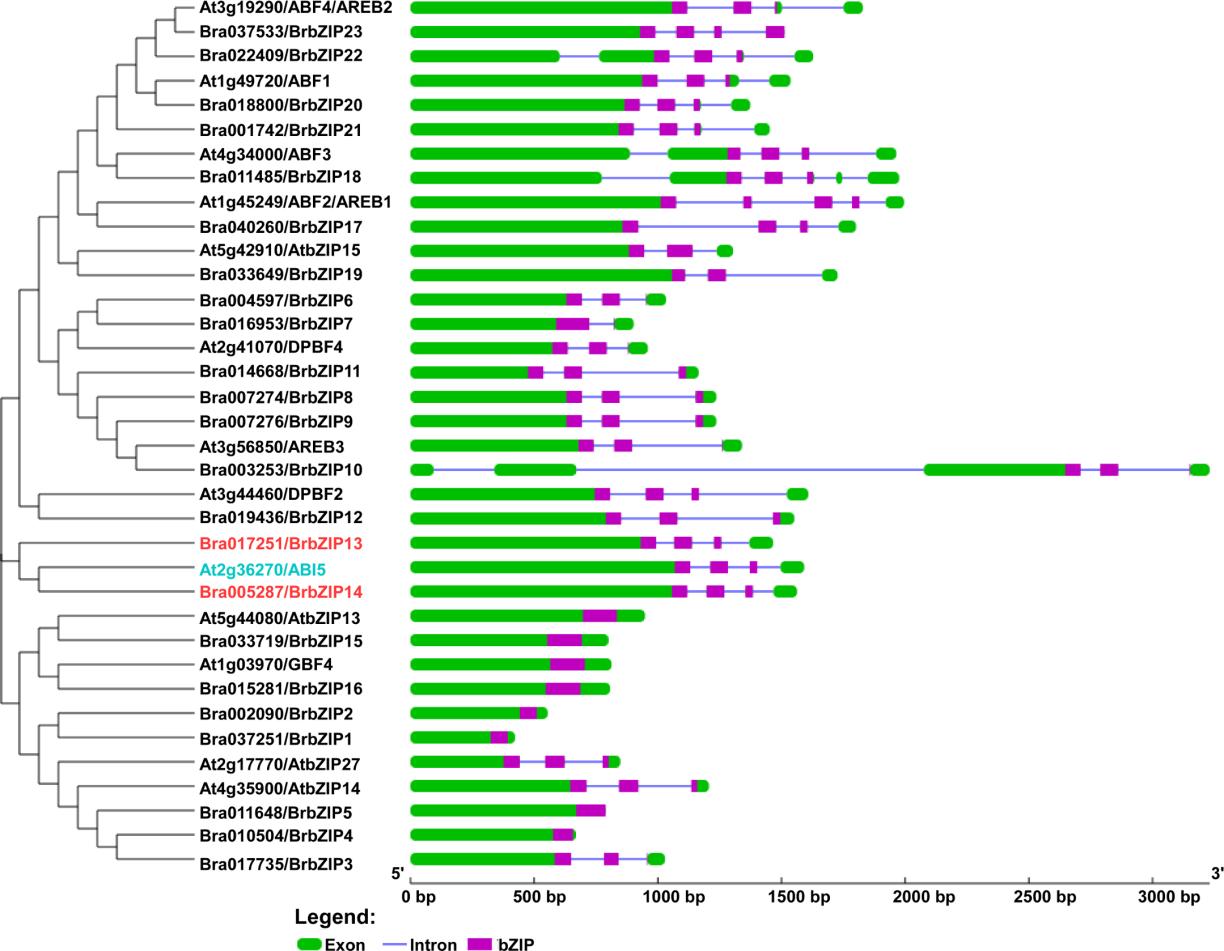
Gene structure of the subfamily A *AtbZIP* and *BrbZIP* genes in Arabidopsis and Chinese cabbage (*Brassica rapa*). Exon/intron organization of subfamily A *AtbZIP* and *BrbZIP* genes was depicted with the online Gene Structure Display Server. The exons and introns are represented by green boxes and blue lines respectively. The purple box denotes the bZIP domain region.

The overall exon/intron profile is an index that determines phylogenetic relationships within a particular gene family from different organisms [38,55]. We also investigated the intron and exon organization of subfamily A *BrbZIP* genes (Fig 2 and S2 Fig). As shown in Fig 2, 17 of subfamily A *BrbZIPs* contained introns. All 17 subfamily A *BrbZIP*s had 1–3 intron/introns within the basic region of the bZIP domain (Fig 2 and S2 Fig). Surprisingly, 10 of these 17 subfamily A *BrbZIP*s members showed a similar gene structure pattern to their Arabidopsis orthologs (Fig 2 and S3 Fig).

### Protein Architecture of Subfamily A BrbZIP Factors

**Fig 3:**
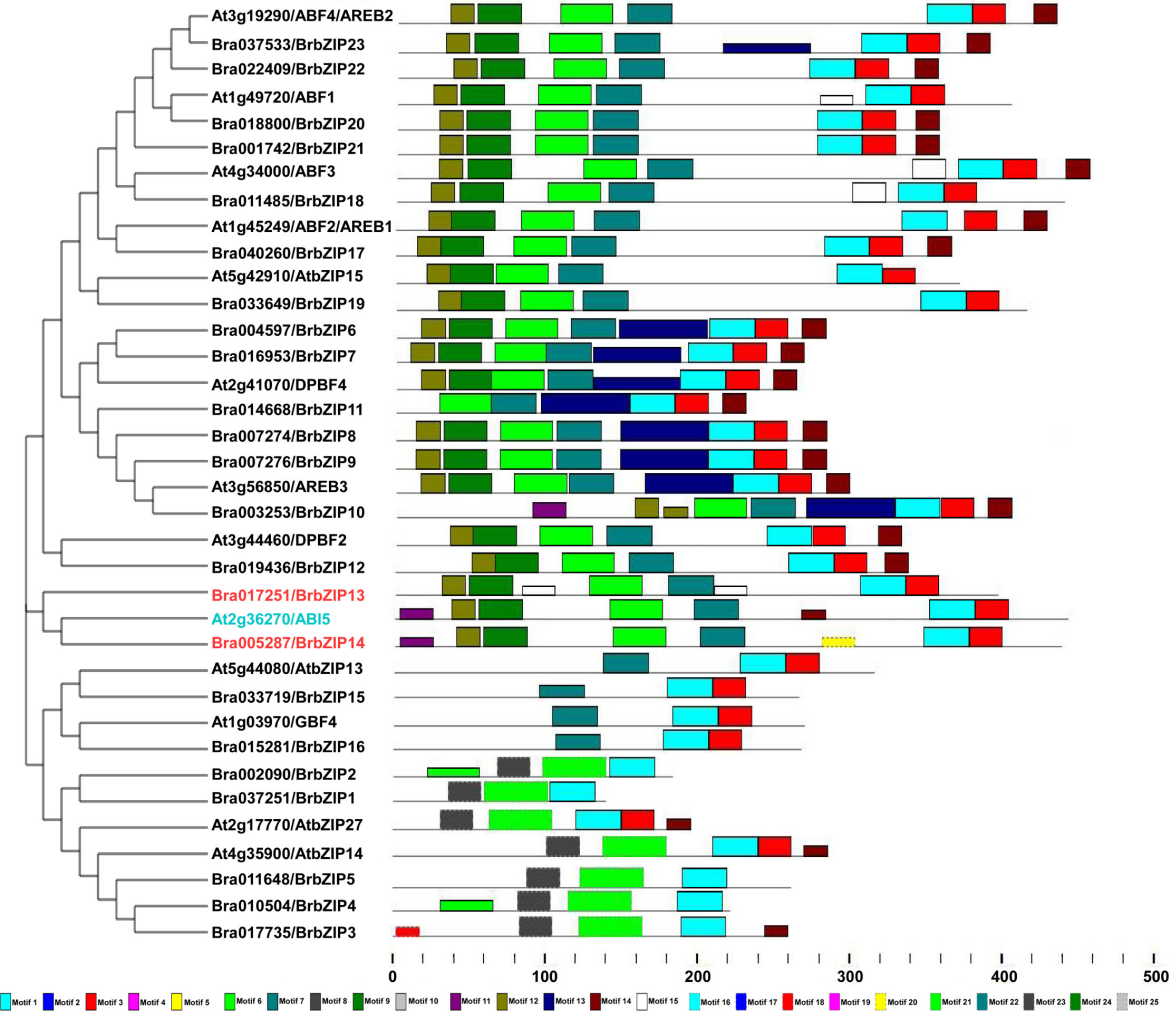
Protein architecture of subfamily A AtbZIP and BrbZIP proteins in Arabidopsis and Chinese cabbage (*Brassica rapa*). The distribution of conserved motifs identified from 23 BrbZIP and 13 AtbZIP proteins of the subfamily A are predicted by the MEME (Multiple Em for Motif Elicitation) tool. Each motif is represented by a number in colored box. See S4 Table for detailed motif information.

To investigate the evolutionary relationships between *Arabidopsis thaliana* and *Brassica rapa*, we found a total of 25 motifs (with *E*-value cutoff <e-1.0)[35], including the conserved bZIP domain (motif 1), in BrbZIPs (S4 Table, S3 Fig). The distribution of motifs in each member of subfamily A bZIPs (13 AtbZIPs and 23 BrbZIPs) was also depicted individually (Fig 3). Motif 3 was shared by most members of this subfamily (31 AtbZIPs and BrbZIPs). Moreover, motif 6 was shared by 27, motif 7 by 29, motif 9 by 24, motif 12 by 25, and motif 14 by 21 members of this subfamily (31 AtbZIPs and BrbZIPs). These conserved motifs were shared by more than 50% members of this subfamily (S5 Table). Interestingly, some of these conserved motifs (6, 7, 9, 12 and 14) were also specifically found in subfamily A (S5 Table). High similarity at the protein architecture level was also observed among orthologs of subfamily A bZIPs between *Arabidopsis thaliana* and *Brassica rapa* (Fig 3). All the motifs found in subfamily A AtbZIPs also appeared in subfamily A BrbZIPs. Some additional motifs (motif 18 and 20) were specifically present in subfamily A BrbZIPs (Fig 3 and S5 Table). The presence of these conserved motifs between subfamily A AtbZIPs and BrbZIPs indicate they may exert similar biological significance in plant signaling pathways.

### Expression Profiles of Subfamily A bZIP Genes in Response to ABA in Chinese Cabbage

**Fig 4:**
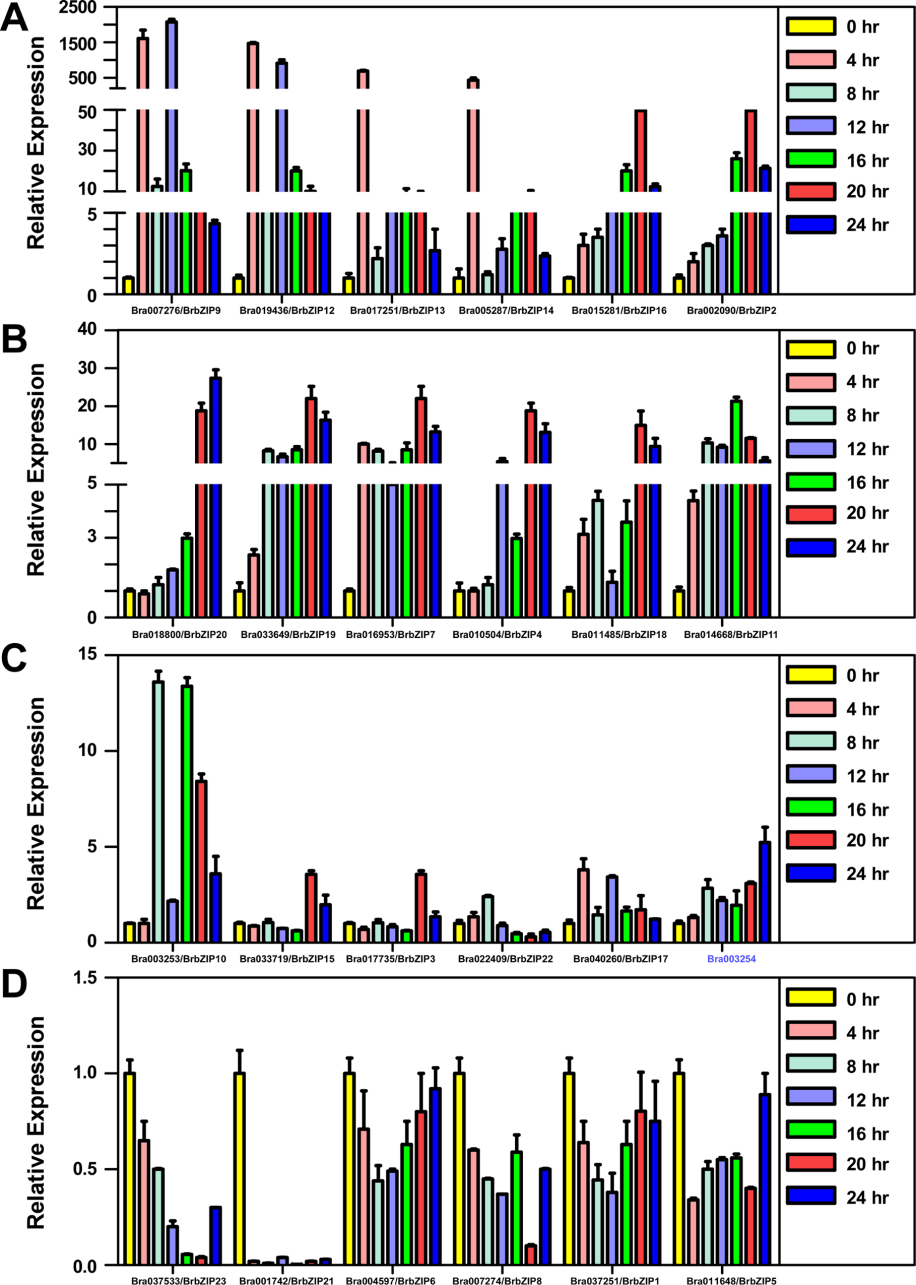
Expression profile of subfamily A *BrbZIP* genes in Chinese cabbage (*Brassica rapa*) after ABA treatment. qRT-PCR analysis of the expression pattern of subfamily A *BrbZIP* genes in response to ABA treatment. Eleven-day-old seedlings were treated with 0.1 mM ABA followed by sampling at 0, 4, 8, 12, 16, 20 and 24 h. The relative expression of the subfamily A *BrbZIP* genes was normalized to the expression of the cabbage *ACTIN2* gene (*BrACTIN2*) and expressed relative to the level in mock-treated seedlings. The expression of Bra003254 after ABA treatment was also determined, which is adjacent to Bra003253/BrbZIP10 (only 1630 bp far from it) and encodes a putative DHHC zinc binding domain like proteins.

To examine the biological significance of subfamily A BrbZIPs in ABA signaling, we determined their expression profile in response to ABA stimulation. Induced expression (more than 2 fold) occurred in many subfamily A *BrbZIPs* starting 4 hr and persisted up to 24 hr following ABA treatment (Fig 4). Interestingly, some members were greatly induced (more than 50 fold Fig 4A) or mildly induced (approximately 30 fold, Fig 4B and 4C).A few of members were not stimulated by ABA with no more than a 2 fold increase at any testing time (Fig 4D). These ABA-induced *BrbZIP* genes are candidates to mediate ABA signaling.

### Sequence Analysis of *BrABI5a* and *BrABI5b* in Chinese Cabbage

Expression of the two putative orthologs of ABI5 in Chinese cabbage, Bra005287/BrbZIP14 and Bra017251/BrbZIP13 (designated as *BrABI5a* and *BrABI5b*, respectively), was strongly induced after ABA treatment (Fig 4). *BrABI5a* and *BrABI5b* encode predicted proteins with 438 and 396 amino acids. The calculated molecular masses of BrABI5a and BrABI5b were 46.2 and 42.3 kDa, and the predicted pI values were 9.22 and 9.48, respectively. Motif analysis showed that BrABI5a and BrABI5b contain nearly all conserved regions of ABI5 and ABI5-like proteins [8,9], such as the four conserved phosphorylation sites including sequences (C1, C2, C3 and C4), a bipartite nuclear localisation signal, and the bZIP domain (Fig 4). Gene architecture of BrABI5a and BrABI5b also displayed high similarity to ABI5 [8,13], which has a large exon at 5’ terminal region followed by three small exons interrupted by three small introns at 3’ terminal region (Fig 2 and S2 Fig). BrABI5a, BrABI5b, BolABI5 and ABI5 were highly conserved (S4 Fig). These findings led us to determine the biological functions of BrABI5a and BrABI5b in response to ABA signalling.

### *BrABI5a* and *BrABI5b* Is Mainly Induced by ABA

**Fig 5:**
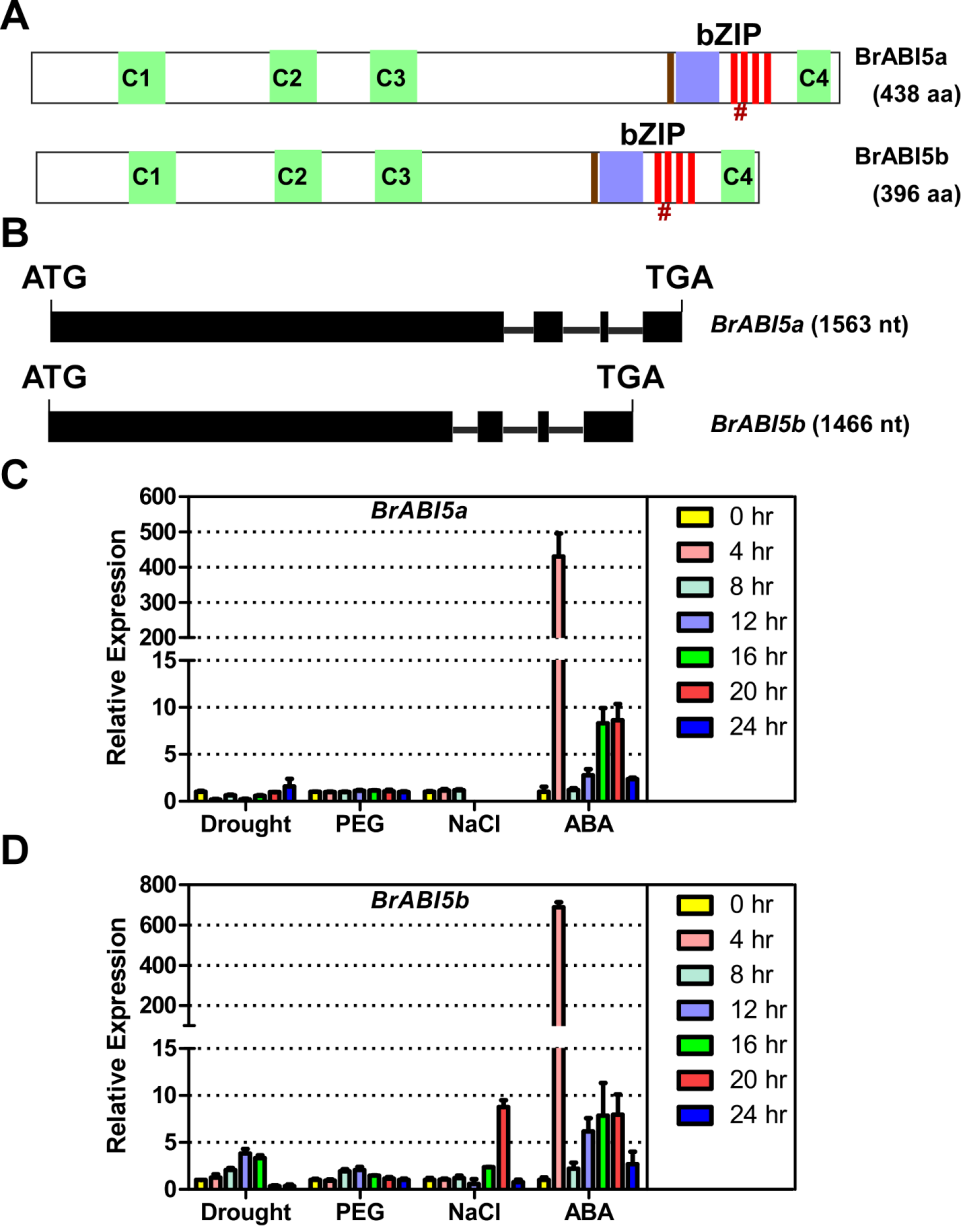
Domain structure and expression pattern of BrABI5a and BrABI5b. (A) Schematic diagram of domains in the BrABI5a and BrABI5b protein. Three N-terminal and one C-terminal conserved sequences (C1, C2, C3 and C4) are shown in the green box, the basic domain is shown in the blue box, the bipartite nuclear localization signal is shown in the black brown rectangle and the Leu residues defining the Leu zipper are shown in the red rectangle, #, the conserved sumoylation site is shown in brown. (B) Exon/intron organization of *BrABI5a* and *BrABI5b* genes. The exons and introns are represented by boxes and lines respectively. (C-D) qRT-PCR analysis of the expression patterns of *BrABI5a* and *BrABI5b* under various environmental stress conditions. The relative expression of *BrABI5a* or *BrABI5b* was normalized to the expression of cabbage *ACTIN2* (*BrACTIN2*) and expressed relative to the level in mock-treated seedlings.

To investigate if BrABI5a and BrABI5b participate in ABA responses, we examined their expression profiles under drought, osmotic, and salt stress, in addition to ABA treatment. ABA-induced expression of *BrABI5a* and *BrABI5b* displayed a similar pattern with the highest expression at 4hr. After a decrease at 8hr, expression returned to basal levels at 12hr and then continually increased until 20hr (Fig 5C and 5D). Moreover, *BrABI5b* expression was induced by drought and salt stress (Fig 5D). However, *BrABI5a* expression was not significantly stimulated by drought (no more than 2 fold) or salt stress (no more than 2 fold) treatments during the testing period (Fig 5C).

### Subcellular Localization of the BrABI5a and BrABI5b

**Fig 6:**
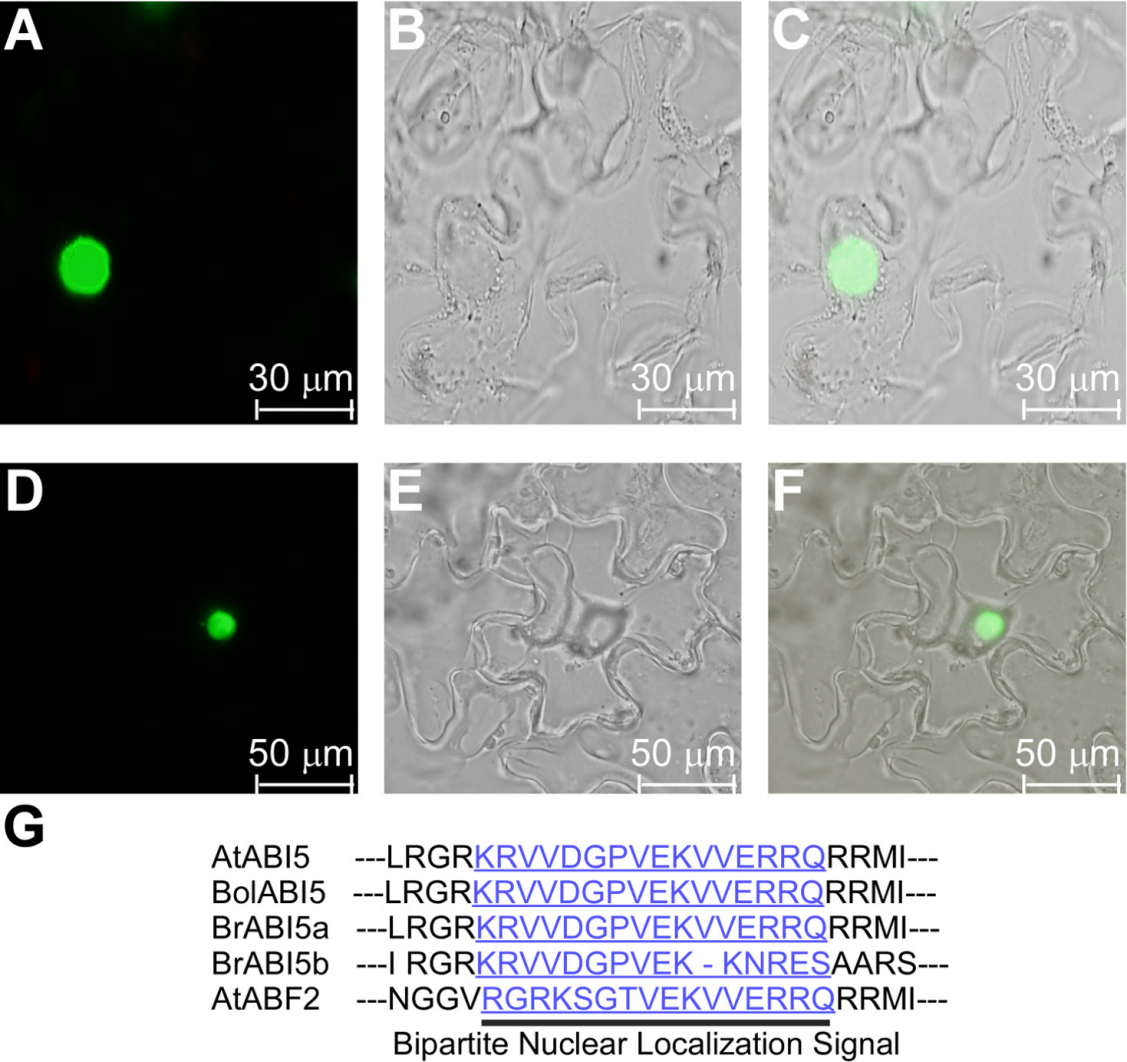
Subcellular localization of BrABI5a and BrABI5b. (A–C) The GFP fluorescence signal of BrABI5a-GFP. (D–F) The GFP fluorescence signal of BrABI5b-GFP. (A, D) Green fluorescence under dark field. (B, E) Cell morphology of the lower epidermis of a tobacco leaf under bright field. (C, F) Overlay of bright-field and green fluorescence signals. (G) The carboxyl-terminal sequence of BrABI5a and BrABI5b are similar to the NLS of the ABF2. The NLS-like motif is underlined and shown in light blue.

As shown in Fig 6G, there is a putative NLS observed at the C-terminus of BrABI5a (amino acids 340 to 373) and BrABI5b (amino acids 298 to 331), respectively. We determined the subcellular localization of BrABI5a and BrABI5b. Both GFP fused BrABI5a and BrABI5b localized exclusively to the nucleus (Fig 6A–6F). These data indicate that BrABI5a and BrABI5b are nuclear-localized proteins.

### Transactivation and DNA-Binding Activity of BrABI5a and BrABI5b

**Fig 7:**
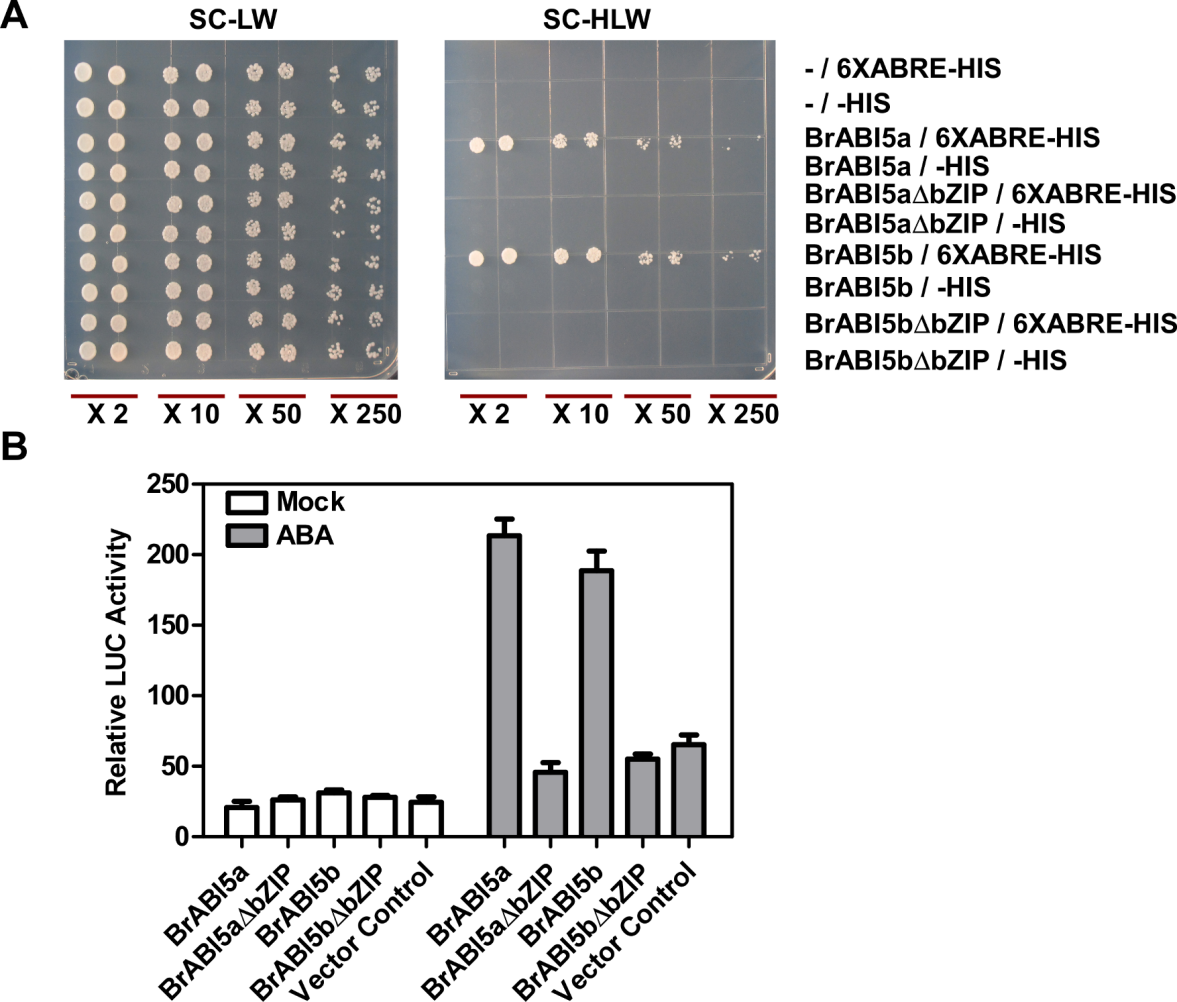
Transactivation activities of BrABI5a and BrABI5b. (A) Yeast one-hybrid analysis of BrABI5a and BrABI5b. Yeast lines yWAM2 expressing the indicated plasmids were grown on synthetic complete medium without Leu and Trp (SC-LW; left) and on synthetic complete medium without Leu, Trp, and His (SC-HLW; right). Yeast cells were incubated until the optical density at 600 nm reached 0.5 and then diluted 2-fold (×2), 10-fold (×10), 50-fold (×50), or 250-fold (×250) and used for assays. (B) Transactivation activity BrABI5a or BrABI5b in Arabidopsis leaf mesophyll protoplasts. Transactivation experiments were performed using protoplasts prepared from Col-0 leaves. Transfected cells were cultured for 16 h without or with 5μM ABA, and relative LUC activity was assayed according to the Dual-Luciferase Reporter Assay Protocol provided by Promega. The empty vector control was also included as a negative control. The values shown are average fLUC (firefly, *Photinus pyralis*, LUC) activities normalized to rLUC (sea pansy, *Renilla reniformis*, LUC) activities. BrABI5aΔbZIP and BrABI5bΔbZIP are forms of BrABI5a and BrABI5b that carries a deletion of the intact C-terminal bZIP region respectively.

We investigated whether BrABI5a and BrABI5b could directly activate ABRE-controlled gene expression. As displayed in Fig 7, BrABI5a and BrABI5b significantly induced *HIS* expression in yeast cells or LUC expression more than 3 fold in Arabidopsis leaf mesophyll protoplasts. Consistent with our previous observations on BolABI5 [9], deletion of the bZIP domain abolished BrABI5a and BrABI5b DNA binding and transactivation activity (Fig 7A and 7B). These results indicate that BrABI5a and BrABI5b possess DNA binding and transactivation activity.

### *BrABI5a* and *BrABI5b* Reverse the Insensitive Phenotype of *abi5-1* to ABA during Seed Germination

**Fig 8:**
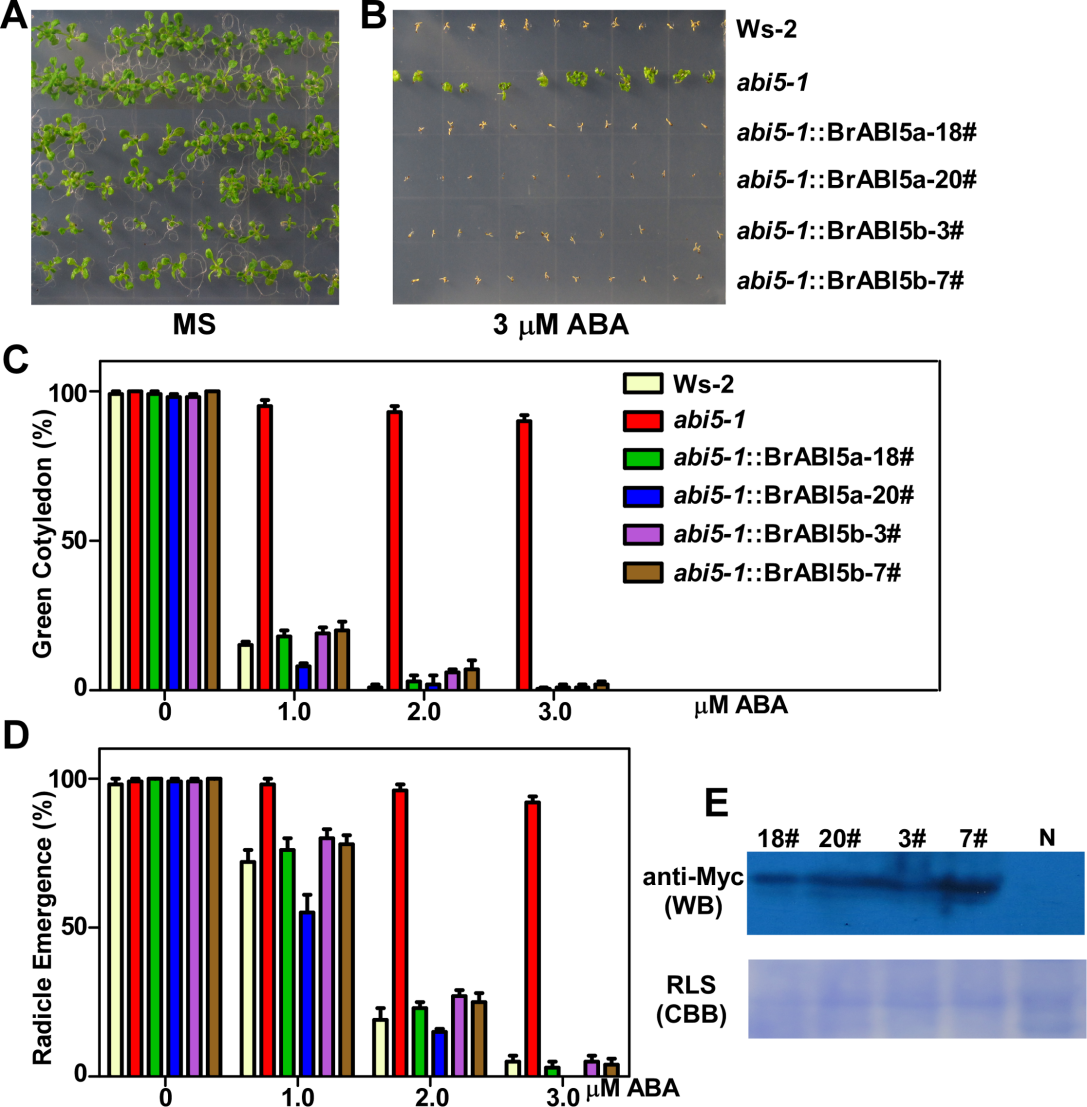
Heterogeneous expression of *BrABI5a* and *BrABI5b* reverse the insensitivity of Arabidopsis *abi5-1* to ABA during seed germination. (A, B) Sensitivity of seed germination to ABA. The seeds of Ws-2, *abi5-1*, and transgenic *abi5-1* lines carrying Myc-tagged BrABI5a or BrABI5b (*abi5-1*::*Myc-BrABI5a* or *abi5-1*::*Myc-BrABI5b*) were germinated on MS medium (A) and MS medium supplemented with 3μM ABA (B) for the indicated days. The emergence rate of green cotyledons (C) and radicle (D) from Ws-2, *abi5-1* and *abi5-1*::*Myc-BrABI5a* or *abi5-1*::*Myc-BrABI5b* transgenic seeds plated on MS supplemented with ABA. Approximately 150 seeds were used in each experiment. Error bars represent SD (seed number > 100). (E) Immunoblots of Myc-BrABI5a or Myc-BrABI5b protein levels in the transgenic *abi5-1* lines (*abi5-1*::*Myc-BrABI5a* or *abi5-1*::*Myc-BrABI5b*). N, transgenic *abi5-1* lines carrying the empty Myc-tagged vector; CBB (Coomassie Brilliant Blue) R250-stained RLS (Rubisco large subunit) served as a loading control.

To determine whether BrABI5a and BrABI5b participate in plant ABA responding, we examined the response of *abi5-1* transgenic lines containing *Myc-BrABI5a* or *Myc-BrABI5b* genes (*abi5-1*::*Myc-BrABI5a* or *abi5-1*::*Myc-BrABI5b*) to ABA. As previously reported [9,13,30], *abi5-1* showed a high germination rate in the presence of ABA (Fig 8A and 8B). In contrast with *abi5-1*, transgenic *abi5-1* lines containing *BrABI5a* or *BrABI5b* were as sensitive to ABA as Ws-2 plants (Fig 8A and 8B). Next, we determined germination frequencies (green cotyledon and radicle emergence ratios) of Ws-2, *abi5-1*, *abi5-1*::*Myc-BrABI5a*, and *abi5-1*::*Myc-BrABI5b* under different ABA concentrations. Similar germination frequencies occurred among *abi5-1*::*Myc-BrABI5a*, *abi5-1*::*Myc-BrABI5b* and Ws-2 plants (Fig 8C and 8D). We also determined Myc-BrABI5a and Myc-BrABI5b protein expression levels in these transgenic plants (Fig 8E). These findings indicate that *BrABI5a* and *BrABI5b* compensates for *abi5* deficiency during seed germination in response to ABA signaling.

## Discussion

Phylogenic analysis reveals that *Brassica rapa* displays a close evolutionary or biological relationship to the model organism *Arabidopsis thaliana* [56,57]. Comparative genomic studies revealed that more than 60% of the genome assemblies between *Arabidopsis thaliana* and *Brassica rapa* are highly conserved [39,40,56,57,58]. Around 93% of the total predicted *Brassica rapa* gene families also appear in *Arabidopsis thaliana* [57]. A previous study found that transcription factors families with a predictable ortholog in *Arabidopsis thaliana* are significantly over retained in *Brassica rapa* [57]. Moreover, genes associated with regulatory networks for environmental stimuli, such as salt, cold, or light, or hormonal responses, such as auxin, brassinosteroid or ABA, in *Arabidopsis thaliana* are also highly retained in *Brassica rapa* [39,40,57]. However, the method used by Hwanng et al., (2014) to classify BrbZIPs increase the difficulty to determine the biological significance of specific subfamily BrbZIPs members [1,3,4,5,35,36,37,38,41]. For example, the putative orthologs of Arabidopsis subfamily A members, ABI5, AREB1/ABF2, AREB2/ABF4 and AREB3 [3,17,18] are divided into group 6A, group 11, group 1A and group 12, respectively [41]. Here, we built a phylogenetic tree with 75 AtbZIPs and 136 BrbZIPs that show all bZIP subfamilies are highly conserved between *Arabidopsis thaliana* and *Brassica rapa* (Fig 1). Moreover, subfamily A members also display a high similarity in their gene and protein architecture (Fig 2 and Fig 3). Functional characterization of two representative genes from subfamily A members further support that the BrbZIPs categorization method employed in our study has increased reliability (Figs 5, 6, 7 and 8). Our findings indicate that a colinear relationship established between AtbZIPs and BrbZIPs provides an advantageous reference to predict and determine the biological function of BrbZIPs in future studies.

Subfamily A bZIP transcription factors participate prominently in ABA signalling and abiotic responses in Arabidopsis [3,10,12,13,14,17,18]. Previous studies demonstrated that several orthologs of ABI5- or ABF-like bZIP transcription factors modulate ABA responses in other plant species [9,27,28,29,30,31,32,33]. We found that many subfamily A orthologs also display high similarities in gene structure and protein architecture between BrbZIPs and AtbZIPs (Fig 2, Fig 3, S2 Fig and S3 Fig). Expression profiles revealed that ABA induces *BrbZIP*s members of subfamily A (Fig 4). Two ABI5 orthologs in *Brassica rapa*, BrABI5a and BrABI5b, have transactivation activity (Fig 7) and positively regulate ABA inhibition of seed germination (Fig 8), as well as ABI5 and other ABI5 orthologs [7,9,13,30,31,32,59].

In the current study, 136 members of bZIP genes are encoded by the *Brassica rapa* genome and are distributed across all 10 chromosomes (S1 Fig). As the A01 to A10 and C01 to C09 represent chromosomes of *Brassica rarpa* and *Brassica oleracea* respectively [41], these genes may be localized inaccurately by Hwanng et al. (2014). Two bZIPs transcription factors that display high similarity to ABI5 was also observed in *Brassica oleracea* (data not shown). In terms of the U’s Triangle, *Brassica rapa* (A genome) and *Brassica oleracea* (C genome) formed the amphidiploid species *Brassica napus* (A and C genomes) during botanical evolution. Four ABI5-like bZIPs transcription factors may exist in *Brassica napus*.

Here, the colinear relationship between AtbZIPs and BrbZIPs will facilitate future study into the biological functions of different BrbZIPs subfamily members in *Brassica rapa* and its close relatives, such as *Brassica oleracea* and *Brassica napus*. In addition, future studies should investigate gene copy number variations of homologous genes and determine their biological significance and/or differences among Arabidopsis, *Brassica rapa*, *Brassica oleracea*, and *Brassica napus*.

## Supporting Information

S1 Fig Chromosomal distribution of bZIP genes in Chinese cabbage (*Brassica rapa*). (DOC)

S2 Fig Gene structure of bZIP genes in Chinese cabbage (*Brassica rapa*). (DOC)

S3 Fig Protein architecture of bZIP proteins in Chinese cabbage (*Brassica rapa*). (DOC)

S4 Fig Phylogenetic analysis of ABI5, ABFs, BrABI5a, BrABI5b and BolABI5. (DOC)

S1 Table DNA primer pairs used for qRT-PCR. (DOC)

S2 Table DNA primer pairs used for constructs generation. (DOC)

S3 Table Overall analysis of bZIP genes in Chinese cabbage (*Brassica rapa*). (DOC)

S4 Table Sequence information of additional conserved motifs identified from 136 bZIP proteins in Chinese cabbage (*Brassica rapa*). (DOC)

S5 Table Summary of additional conserved motifs predicted in subfamily A bZIP proteins of Chinese cabbage (*Brassica rapa*) and Arabidopsis. (DOC)
