# Supplementary material for: Genome-Wide Analysis of the bZIP Gene Family Identifies Two ABI5-Like bZIP Transcription Factors, BrABI5a and BrABI5b, as Positive Modulators of ABA Signalling in Chinese Cabbage
Source: PLoS One. 2016 Jul 14;11(7):e0158966. doi: 10.1371/journal.pone.0158966 (PMC4944949; doi:10.1371/journal.pone.0158966)
Supplement: S1 Fig — (DOC) [file pone.0158966.s001.doc]

S1 Fig


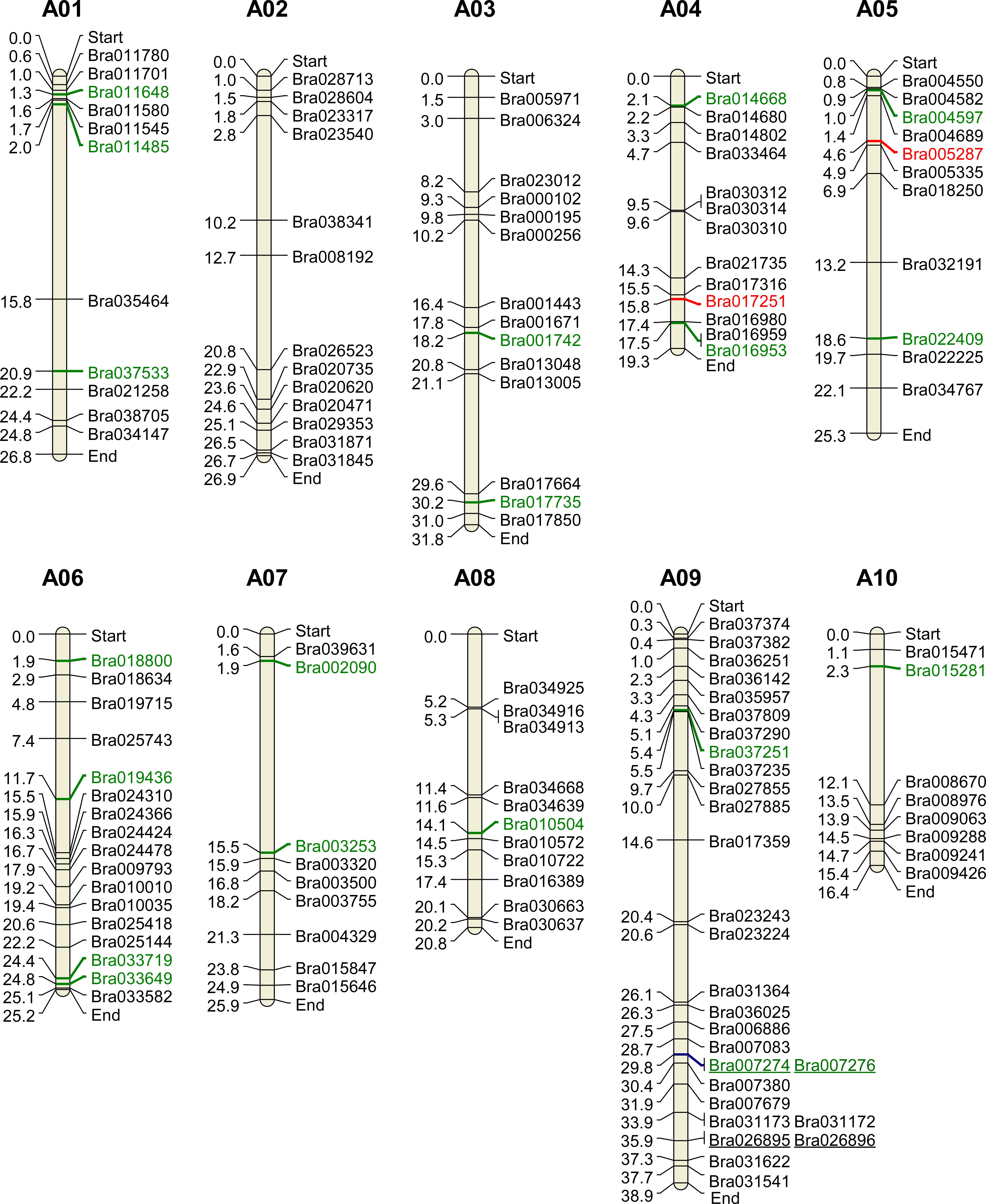


S1 Fig. Chromosomal distribution of bZIP TF genes in Chinese cabbage (*Brassica rapa*).

Chromosome numbers are indicated at the top of each bar and chromosomal distances are given in Mbp at the left of each bar. The two ABI5-like orthologs are red labeled, the other members of subfamily A *BrbZIP* genes are labeled in green, and tandem-duplicated *BrbZIP* genes are underlined. Bra040260 gene was finally anchored on Scaffold00019, which has not yet been mapped on any chromosome.
