# Supplementary material for: Genome-Wide Analysis of the bZIP Gene Family Identifies Two ABI5-Like bZIP Transcription Factors, BrABI5a and BrABI5b, as Positive Modulators of ABA Signalling in Chinese Cabbage
Source: PLoS One. 2016 Jul 14;11(7):e0158966. doi: 10.1371/journal.pone.0158966 (PMC4944949; doi:10.1371/journal.pone.0158966)
Supplement: S2 Fig — (DOC) [file pone.0158966.s002.doc]

S2 Fig


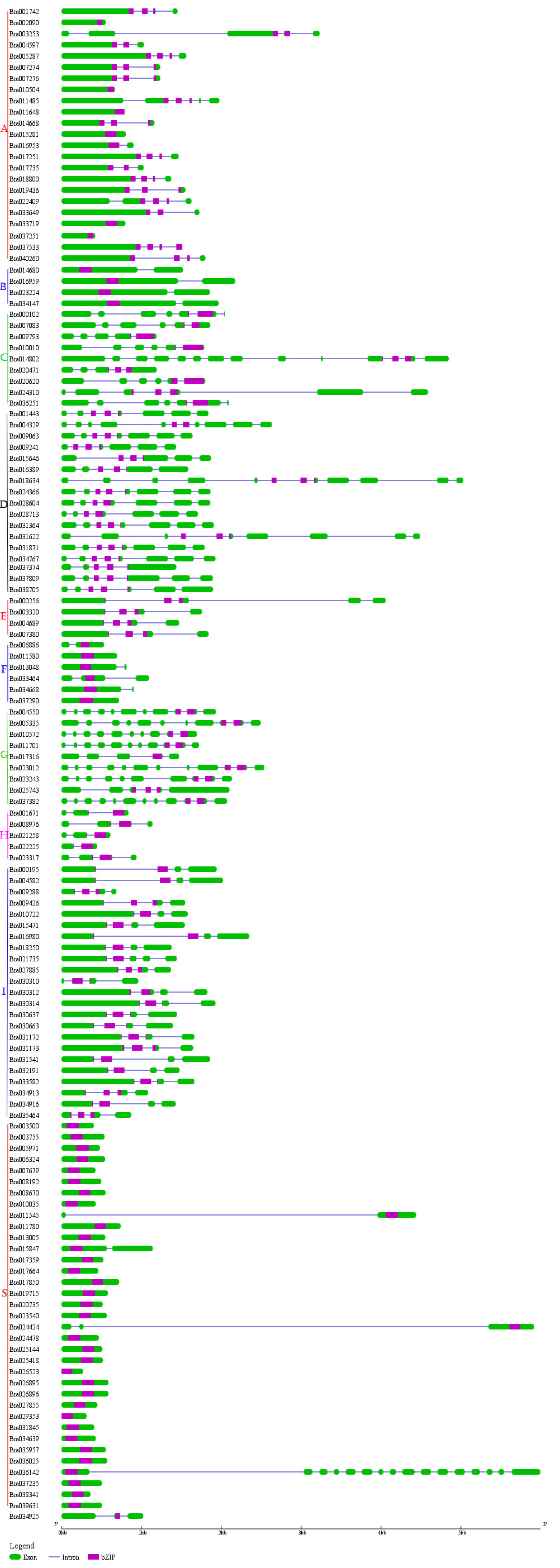


S2 Fig. Gene structure of bZIP genes in Chinese cabbage (*Brassica rapa*).

Exon/intron organization of BrbZIPs was depicted with the online Gene Structure Display Server. The exons and introns are represented by green boxes and blue lines respectively. The purple box denotes the bZIP domain region.
