# Supplementary material for: Genome-Wide Analysis of the bZIP Gene Family Identifies Two ABI5-Like bZIP Transcription Factors, BrABI5a and BrABI5b, as Positive Modulators of ABA Signalling in Chinese Cabbage
Source: PLoS One. 2016 Jul 14;11(7):e0158966. doi: 10.1371/journal.pone.0158966 (PMC4944949; doi:10.1371/journal.pone.0158966)
Supplement: S3 Fig — (DOC) [file pone.0158966.s003.doc]

S3 Fig.


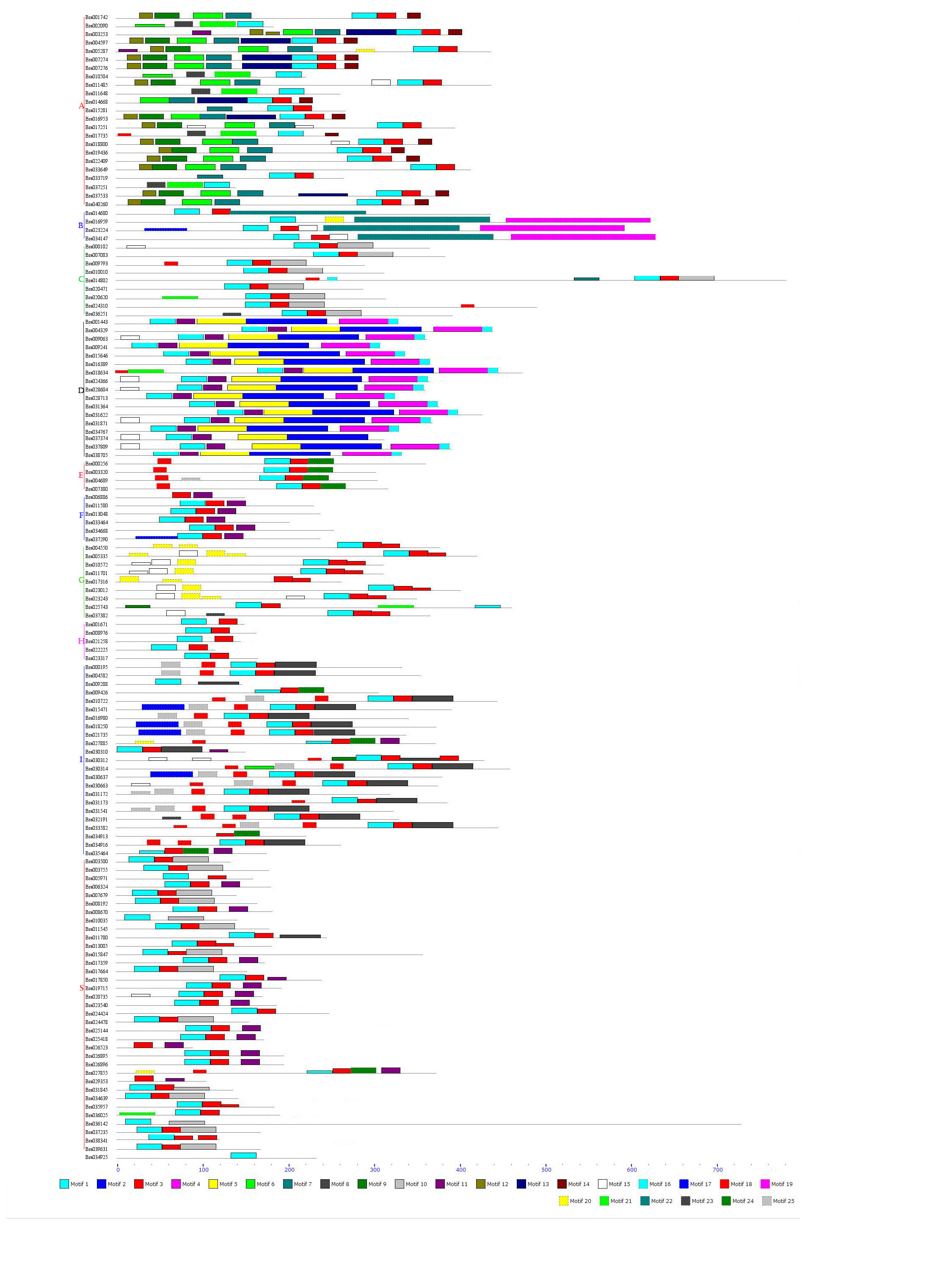


S3 Fig. Protein architecture of bZIP proteins in Chinese cabbage (*Brassica rapa*).

Summary for the distribution of conserved motifs identified from a total of 136 BrbZIPs by the MEME (Multiple Em for Motif Elicitation) tool. Each motif is represented by a number in colored box. See Supplementary Table S4 for detailed motif information.
