# Supplementary material for: Genome-Wide Analysis of the bZIP Gene Family Identifies Two ABI5-Like bZIP Transcription Factors, BrABI5a and BrABI5b, as Positive Modulators of ABA Signalling in Chinese Cabbage
Source: PLoS One. 2016 Jul 14;11(7):e0158966. doi: 10.1371/journal.pone.0158966 (PMC4944949; doi:10.1371/journal.pone.0158966)
Supplement: S4 Fig — (DOC) [file pone.0158966.s004.doc]

S4 Fig.


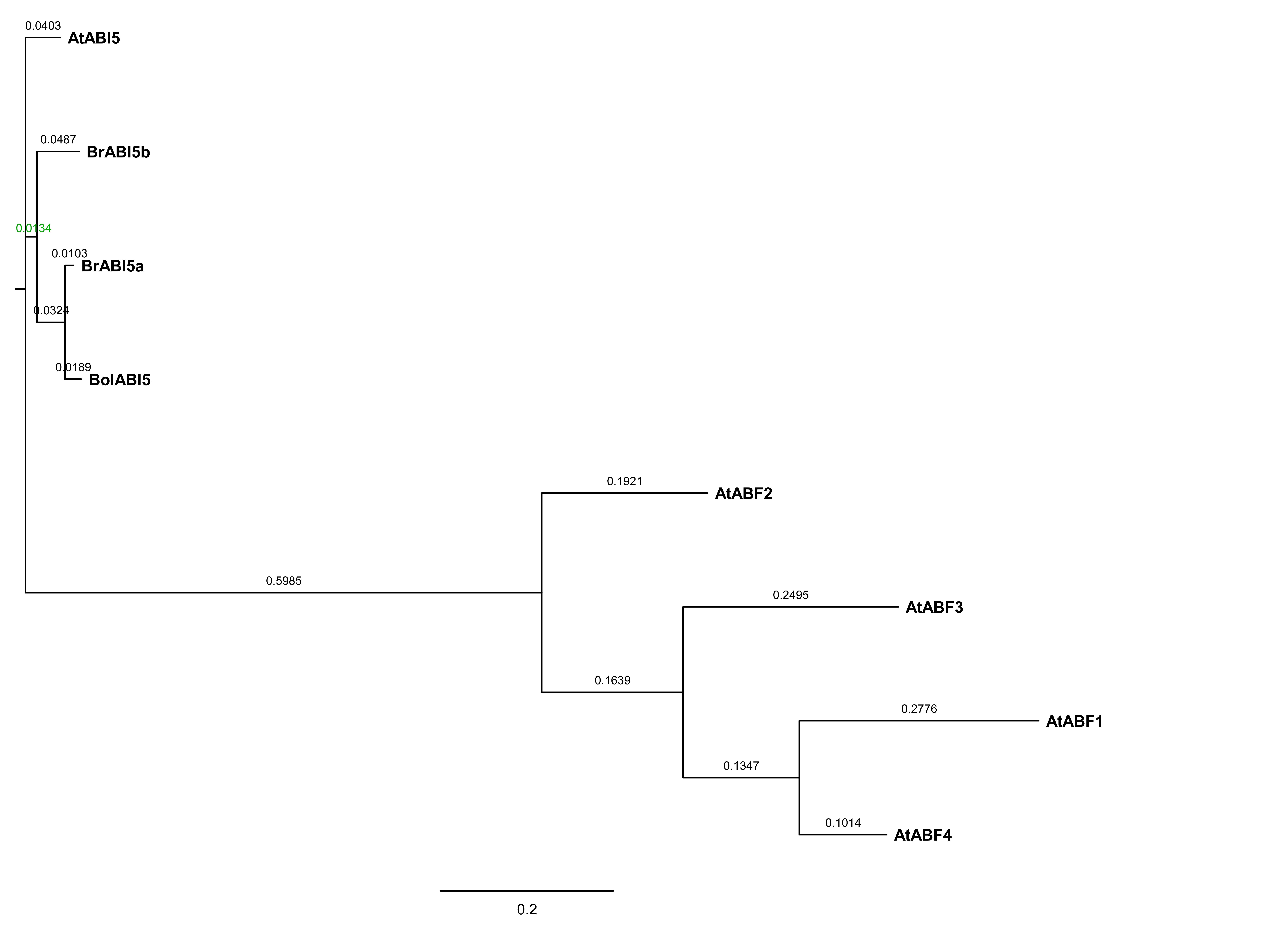


S4 Fig. Phylogenetic analysis of AtABI5, AtABFs, BrABI5a, BrABI5b and BolABI5.

The protein sequences were aligned by the MUSCLE tool and the maximum likelihood tree was generated using MEGA 5.0.
