# Supplementary material for: Genome-Wide Analysis of the bZIP Gene Family Identifies Two ABI5-Like bZIP Transcription Factors, BrABI5a and BrABI5b, as Positive Modulators of ABA Signalling in Chinese Cabbage
Source: PLoS One. 2016 Jul 14;11(7):e0158966. doi: 10.1371/journal.pone.0158966 (PMC4944949; doi:10.1371/journal.pone.0158966)
Supplement: S1 Table — (DOC) [file pone.0158966.s005.doc]

S1 Table. DNA primer pairs used for qRT-PCR.

| S1 Table. DNA Primer Pairs Used for qRT-PCR. | |
| --- | --- |
| Name | Sequence (5'---3') |
| Bra033649qF | CCAACTAACGGTGTGAAC |
| Bra033649qR | GCTGCTTCTGAATCTGAG |
| Bra007274qF | GGTAATCAGCCATCTTCC |
| Bra007274qR | GTCCTCAAGCGTCATTTC |
| Bra007276qF | TGGCTGTCAATGAAGGTCTC |
| Bra007276qR | GTCCTGCTGAATGTCTTTCC |
| Bra014668qF | GAGCCTACGCTTGGTGAAATG |
| Bra014668qR | CAGCCTGAGGTATGTTCTGTC |
| Bra003253qF | ACACAGCCTCGGTCATTCTTTC |
| Bra003253qR | TCCTTCCTGGAGTTTGCGTATC |
| Bra003254qF | TCTCTGTCGCTTGGATACTC |
| Bra003254qR | GTTCTACCGCTAACCCATTC |
| Bra019436qF | TACTCGGGTATGGGAGAATC |
| Bra019436qR | TAATCATCCGTCGCTGTC |
| Bra040260qF | AGTGTCAGCAGATGGGATAG |
| Bra040260qR | CTTCTTTGCCTCCTCTCAAC |
| Bra022409qF | GGTGGGTATGGTCAACAAG |
| Bra022409qR | TAAGGAACTGGCGACAGAG |
| Bra001742qF | AACAACGGTGCTGCTGAAGG |
| Bra001742qR | TGATGCTGCTGCTGTGGATG |
| Bra011485qF | TGGTGTGGTTAGGGAAGAG |
| Bra011485qR | CGGCTGAGTTTGAGTTGTC |
| Bra037533qF | GGTTCCTTACGTGCTTAACC |
| Bra037533qR | CCATCATCTCAGCCTGTTTC |
| Bra018800qF | GTGGCATCAACTTCTCCTAGTG |
| Bra018800qR | AAGGTGTAAGCCTGCTTTCG |
| Bra033719qF | AGTGATGATGGAGCCTTTGG |
| Bra033719qR | CAACTTGGTAAGCGTGCTTC |
| Bra015281qF | GACTTCTTAGCGCAAGCAG |
| Bra015281qR | TTCTCCTCGTCGATCCTTC |
| Bra004597qF | TTGTGGAGAGGGTTGTAGAG |
| Bra004597qR | GTTCACTTGGCAGGATCTTC |
| Bra016953qF | CAACGGCTCTGATGATGAGTG |
| Bra016953qR | TCCTAGCTCGTGAACGTGATG |
| Bra011648qF | GACATCCACCACCTAAACC |
| Bra011648qR | GGCCATTAGAGGAAGAACC |
| Bra010504qF | GAGTCTCTGCTCCAAACAAG |
| Bra010504qR | GGTGGTTAAGGTGGTGAATG |
| Bra017735qF | GAGCTGGCTTTGAGTTTC |
| Bra017735qR | AAGTCGGTGTAACCAGAG |
| Bra002090qF | CCTCCTGAAACTGTTCTC |
| Bra002090qR | GCTTATGCCTTCTGTCTC |
| Bra037251qF | CTCCTCCTGCAACTGTTATC |
| Bra037251qR | AGACACGTTCCTGTTTCC |
| Bra005287qF | GCTAATAACGGCGTTGGTGAG |
| Bra005287qR | TACACCCACCGAGAAAGAAGG |
| Bra017251qF | ACACTTCCATCTCCGCTTTG |
| Bra017251qR | ATGACCACCGCCTTTATGAC |
| BrACTIN2-qF | GCAGACCGTATGAGCAAAG |
| BrACTIN2-qR | GCTGAGGGAAGCAAGAATG |
