# Supplementary material for: Genome-Wide Analysis of the bZIP Gene Family Identifies Two ABI5-Like bZIP Transcription Factors, BrABI5a and BrABI5b, as Positive Modulators of ABA Signalling in Chinese Cabbage
Source: PLoS One. 2016 Jul 14;11(7):e0158966. doi: 10.1371/journal.pone.0158966 (PMC4944949; doi:10.1371/journal.pone.0158966)
Supplement: S2 Table — (DOC) [file pone.0158966.s006.doc]

S2 Table. DNA primer pairs used for constructs generation.

| S2 Table. DNA Primer Pairs Used for Constructs Generation. | |
| --- | --- |
| Name | Sequence (5'---3') |
| BrABI5a-GFP | CGCGGATCCATGGTGGGCAGAGAAACAGAG |
|  | ACGCGTCGACGAGAGGGCAACTAGGGTTCCTC |
| BrABI5b-GFP | CGCGGATCCATGATGTCTGGACGAGAAGTAG |
|  | ACGCGTCGACGAGCGGACAACTCGGATTCC |
| pPC86-BrABI5a | ACGCGTCGACCATGGTGGGCAGAGAAACAGAG |
|  | CCGGAATTCTCAGAGAGGGCAACTAGGGTTC |
| pPC86-BrABI5b | ACGCGTCGACCATGATGTCTGGACGAGAAGTAG |
|  | CCGGAATTCTCAGACGAGCGGACAACTCG |
| pPC86-BrABI5aΔbZIP | ACGCGTCGACCATGGTGGGCAGAGAAACAGAG |
|  | CCGGAATTCCTCCACTACTTTCTCCAC |
| pPC86-BrABI5bΔbZIP | ACGCGTCGACCATGATGTCTGGACGAGAAGTAG |
|  | CCGGAATTCCTCAACCACCTTCTCTACC |
| 6myc-BrABI5a | CGCGGATCCATGGTGGGCAGAGAAACAGAG |
|  | ACGCGTCGACGAGAGGGCAACTAGGGTTCCTC |
| 6myc-BrABI5b | CGCGGATCCATGATGTCTGGACGAGAAGTAG |
|  | ACGCGTCGACGAGCGGACAACTCGGATTCC |
| 6myc-BrABI5aΔbZIP | CGCGGATCCATGGTGGGCAGAGAAACAGAG |
|  | ACGCGTCGACCTCCACTACTTTCTCCAC |
| 6myc-BrABI5bΔbZIP | CGCGGATCCATGATGTCTGGACGAGAAGTAG |
|  | ACGCGTCGACCTCAACCACCTTCTCTACC |
