# Supplementary material for: Genome-Wide Analysis of the bZIP Gene Family Identifies Two ABI5-Like bZIP Transcription Factors, BrABI5a and BrABI5b, as Positive Modulators of ABA Signalling in Chinese Cabbage
Source: PLoS One. 2016 Jul 14;11(7):e0158966. doi: 10.1371/journal.pone.0158966 (PMC4944949; doi:10.1371/journal.pone.0158966)
Supplement: S3 Table — (DOC) [file pone.0158966.s007.doc]

S3 Table. Overall analysis of bZIP genes in Chinese cabbage (*Brassica rapa*).

| S3 Table. Overall analysis of bZIP genes in Chinese cabbage (*Brassica rapa*). | | | | | | | | | |
| --- | --- | --- | --- | --- | --- | --- | --- | --- | --- |
| Gene | Accession number | ORF (bp) | Chr | Protein | | | | Subfamily | Orthologs in Arabdiopsis |
|  |  |  |  | Length (aa) | MW (D) | pI | Position of bZIP domain |  |  |
| BrbZIP1 | Bra037251 | 423 | A09 | 140 | 15974.8 | 9.17 | R-109～R-131 | A |  |
| BrbZIP2 | Bra002090 | 555 | A07 | 184 | 20890.34 | 9.49 | R-148～C-170 | A | At2g17770,AtbZIP27 |
| BrbZIP3 | Bra017735 | 780 | A03 | 259 | 28741.86 | 10.1 | R-195～L-237 | A |  |
| BrbZIP4 | Bra010504 | 669 | A08 | 222 | 24787.61 | 9.78 | R-193～L-219 | A | At4g35900,AtbZIP14 |
| BrbZIP5 | Bra011648 | 789 | A01 | 262 | 29434.98 | 9.34 | R-196～C-218 | A | At4g35900,AtbZIP14 |
| BrbZIP6 | Bra004597 | 846 | A05 | 281 | 31564.91 | 9.04 | R-211～E-256 | A | At2g41070,AtbZIP12,DPBF4 |
| BrbZIP7 | Bra016953 | 804 | A04 | 267 | 30038.09 | 7.98 | R-197～E-242 | A | At2g41070,AtbZIP12,DPBF4 |
| BrbZIP8 | Bra007274 | 849 | A09 | 282 | 31258.3 | 9.25 | R-211～P-265 | A | At3g56850,AtbZIP66,AREB3 |
| BrbZIP9 | Bra007276 | 849 | A09 | 282 | 31374.45 | 9.25 | R-211～P-265 | A | At3g56850,AtbZIP66,AREB3 |
| BrbZIP10 | Bra003253 | 1212 | A07 | 403 | 44951.79 | 10.12 | R-333～E-378 | A | At3g56850,AtbZIP66,AREB3 |
| BrbZIP11 | Bra014668 | 690 | A04 | 229 | 25486.9 | 9.12 | R-159～P-213 | A | At3g56850,AtbZIP66,AREB3 |
| BrbZIP12 | Bra019436 | 1011 | A06 | 336 | 37973.05 | 7.4 | R-264～K-318 | A | At3g44460,AtbZIP67,DPBF2 |
| BrbZIP13 | Bra017251 | 1191 | A04 | 396 | 42302.05 | 9.48 | R-311～Q-365 | A | At2g36270,AtbZIP39,ABI5 |
| BrbZIP14 | Bra005287 | 1317 | A05 | 438 | 46222.24 | 9.22 | R-353～Q-407 | A | At2g36270,AtbZIP39,ABI5 |
| BrbZIP15 | Bra033719 | 801 | A06 | 266 | 29817.58 | 5.81 | Q-185～E-231 | A |  |
| BrbZIP16 | Bra015281 | 807 | A10 | 268 | 30513.88 | 5.54 | Q-183～E-229 | A | At1g03970,AtbZIP40,GBF4 |
| BrbZIP17 | Bra040260 | 1095 | Scaffold  000193 | 364 | 39043.15 | 9.3 | R-287～Q-341 | A |  |
| BrbZIP18 | Bra011485 | 1320 | A01 | 439 | 48105.4 | 8.61 | R-335～Q-389 | A | At4g34000,AtbZIP37,ABF3 |
| BrbZIP19 | Bra033649 | 1245 | A06 | 414 | 45707.53 | 9.39 | R-353～Q-394 | A | At5g42910,AtbZIP15 |
| BrbZIP20 | Bra018800 | 1107 | A06 | 368 | 40298.99 | 9.23 | R-289～K-341 | A |  |
| BrbZIP21 | Bra001742 | 1068 | A03 | 355 | 39295.81 | 9.32 | R-281～K-333 | A |  |
| BrbZIP22 | Bra022409 | 1065 | A05 | 453 | 38106.58 | 9.44 | R-276～K-328 | A | At1g49720,AtbZIP35,ABF1 |
| BrbZIP23 | Bra037533 | 1167 | A01 | 388 | 422288.24 | 9.62 | R-310～K-362 | A | At1g49720,AtbZIP35,ABF1 |
| BrbZIP24 | Bra014680 | 1317 | A04 | 438 | 48850.33 | 6.48 | R-75～A-126 | B |  |
| BrbZIP25 | Bra016959 | 1875 | A04 | 624 | 68527.89 | 5.29 | R-188～A-238 | B | At2g40950,AtbZIP17 |
| BrbZIP26 | Bra034147 | 1890 | A01 | 629 | 68736.54 | 5.86 | K-191～A-242 | B | At3g10800,AtbZIP28 |
| BrbZIP27 | Bra023224 | 1782 | A09 | 593 | 65165.16 | 6.3 | K-155～A-206 | B | At3g10800,AtbZIP28 |
| BrbZIP28 | Bra020471 | 870 | A02 | 289 | 3172029 | 5.16 | R-134～R-185 | C | At5g24800,AtbZIP9，BZO2H2 |
| BrbZIP29 | Bra009793 | 876 | A06 | 291 | 31933.48 | 6.89 | T-203～S-280 | C | At5g24800,AtbZIP9，BZO2H2 |
| BrbZIP30 | Bra010010 | 945 | A06 | 314 | 34426.26 | 8.53 | T-222～S-310 | C |  |
| BrbZIP31 | Bra020620 | 948 | A02 | 315 | 34512.65 | 8.75 | T-224～S-311 | C | At5g28770,AtbZIP63，BZO2H3 |
| BrbZIP32 | Bra036251 | 1185 | A09 | 394 | 43201.74 | 5.15 | T-267～H-368 | C | At4g02640,AtbZIP10，BZO2H1 |
| BrbZIP33 | Bra000102 | 1101 | A03 | 366 | 39536.73 | 4.83 | T-280～E-348 | C |  |
| BrbZIP34 | Bra014802 | 2352 | A04 | 783 | 85613.87 | 8.2 | R-613～D-664 | C |  |
| BrbZIP35 | Bra007083 | 1155 | A09 | 384 | 42176.85 | 6.14 | T-303～P-339 | C | At3g54620,AtbZIP25 |
| BrbZIP36 | Bra024310 | 1476 | A06 | 491 | 56052.36 | 7.14 | V-155～D-208 | C | At3g54620,AtbZIP25 |
| BrbZIP37 | Bra015646 | 1020 | A07 | 339 | 38888.36 | 8.4 | K-63～S-114 | D |  |
| BrbZIP38 | Bra031364 | 1137 | A09 | 378 | 42825.1 | 6.09 | K-92～L-140 | D | At1g22070,AtbZIP22，TGA3 |
| BrbZIP39 | Bra016389 | 1107 | A08 | 368 | 41719.85 | 6.84 | K-89～L-137 | D | At1g77920,AtbZIP50 |
| BrbZIP40 | Bra009063 | 1095 | A10 | 364 | 41816.38 | 6.93 | K-80～S-131 | D | At5g10030,AtbZIP57，OBF4，TGA4 |
| BrbZIP41 | Bra028604 | 1089 | A02 | 362 | 41614.27 | 7.56 | K-78～G-129 | D |  |
| BrbZIP42 | Bra031871 | 1113 | A02 | 370 | 42230.02 | 8.18 | K-86～G-137 | D |  |
| BrbZIP43 | Bra024366 | 1104 | A06 | 367 | 41950.61 | 7.59 | K-83～G-134 | D |  |
| BrbZIP44 | Bra037809 | 1179 | A09 | 392 | 44724.73 | 7.84 | K-81～G-132 | D |  |
| BrbZIP45 | Bra037374 | 945 | A09 | 314 | 36483.84 | 8.41 | K-65～G-116 | D |  |
| BrbZIP46 | Bra004329 | 1326 | A07 | 441 | 49720 | 5.9 | K-154～Q-200 | D | At1g68640,AtbZIP46，PAN |
| BrbZIP47 | Bra031622 | 1287 | A09 | 428 | 47734.36 | 8.77 | K-125～G-177 | D | At1g08320,AtbZIP21 |
| BrbZIP48 | Bra018634 | 1428 | A06 | 475 | 53099.94 | 7.57 | K-172～G-224 | D | At1g08320,AtbZIP21 |
| BrbZIP49 | Bra028713 | 981 | A02 | 326 | 36672.17 | 8.85 | K-42～S-94 | D | At5g06960,AtbZIP26，OBF5，TGA5 |
| BrbZIP50 | Bra009241 | 933 | A10 | 310 | 34639.37 | 9.56 | K-26～S-78 | D |  |
| BrbZIP51 | Bra034767 | 996 | A05 | 331 | 36848.5 | 8.64 | K-47～S-99 | D | At3g12250,AtbZIP45，TGA6 |
| BrbZIP52 | Bra001443 | 996 | A03 | 331 | 36829.48 | 8.96 | K-47～S-99 | D | At3g12250,AtbZIP45，TGA6 |
| BrbZIP53 | Bra038705 | 1005 | A01 | 334 | 37121.9 | 8.14 | K-50～S-102 | D | At3g12250,AtbZIP45，TGA6 |
| BrbZIP54 | Bra004689 | 921 | A05 | 306 | 34404.84 | 6.82 | R-175～R-225 | E | At2g42380,AtbZIP34 |
| BrbZIP55 | Bra000256 | 1089 | A03 | 362 | 40968.84 | 6.14 | R-181～R-231 | E | At2g42380,AtbZIP34 |
| BrbZIP56 | Bra003320 | 915 | A07 | 304 | 34093.31 | 6.27 | R-180～R-230 | E |  |
| BrbZIP57 | Bra007380 | 957 | A09 | 318 | 35442.57 | 6.27 | R-195～R-245 | E | At3g58120,AtbZIP61 |
| BrbZIP58 | Bra006886 | 456 | A09 | 151 | 17177.83 | 5.44 | K-56～M-91 | F | At3g51960 |
| BrbZIP59 | Bra033464 | 612 | A04 | 203 | 22954.73 | 6.46 | N-65～S-103 | F |  |
| BrbZIP60 | Bra013048 | 720 | A03 | 239 | 25824 | 6.67 | N-78～V-124 | F |  |
| BrbZIP61 | Bra037290 | 720 | A09 | 239 | 26217.36 | 5.63 | N-76～V-132 | F |  |
| BrbZIP62 | Bra034668 | 768 | A08 | 255 | 27591.62 | 5.74 | K-95～S-147 | F |  |
| BrbZIP63 | Bra011580 | 696 | A01 | 231 | 25372.02 | 5.87 | E-83～S-136 | F | At4g35040 |
| BrbZIP64 | Bra025743 | 1389 | A06 | 462 | 51055.79 | 5.03 | L-152～Q-198 | G | At1g19490，AtbZIP62 |
| BrbZIP65 | Bra011701 | 942 | A01 | 313 | 33596.94 | 5.87 | R-223～C-273 | G |  |
| BrbZIP66 | Bra010572 | 942 | A08 | 313 | 33772.24 | 6.37 | R-226～C-276 | G | At4g36730，AtbZIP41，GBF1 |
| BrbZIP67 | Bra037382 | 1104 | A09 | 367 | 39607.68 | 6.56 | R-254～N-303 | G | At4g01120，AtbZIP54，GBF2 |
| BrbZIP68 | Bra004550 | 1140 | A05 | 379 | 40806.13 | 9.36 | R-266～N-317 | G | At2g46270，AtbZIP55，GBF3 |
| BrbZIP69 | Bra023012 | 1212 | A03 | 403 | 43509.31 | 6.36 | E-299～L-355 | G | At1g32150，AtbZIP68 |
| BrbZIP70 | Bra023243 | 1059 | A09 | 352 | 37685.59 | 5.94 | R-250～Y-300 | G |  |
| BrbZIP71 | Bra017316 | 795 | A04 | 264 | 28405.04 | 4.85 | L-181～N-219 | G |  |
| BrbZIP72 | Bra005335 | 1269 | A05 | 422 | 45351.29 | 5.31 | R-320～D-371 | G |  |
| BrbZIP73 | Bra023317 | 501 | A02 | 166 | 18100.86 | 9.53 | R-87～H-138 | H |  |
| BrbZIP74 | Bra008976 | 495 | A10 | 164 | 18064.86 | 9.31 | R-88～Q-139 | H |  |
| BrbZIP75 | Bra022225 | 351 | A05 | 116 | 13260.73 | 8.14 | K-50～N-98 | H |  |
| BrbZIP76 | Bra021258 | 441 | A01 | 146 | 16444.07 | 7.18 | L-79～N-128 | H |  |
| BrbZIP77 | Bra001671 | 453 | A03 | 150 | 16968.74 | 6.83 | L-84～N-133 | H | At3g17609，AtbZIP64，HYH |
| BrbZIP78 | Bra030312 | 1293 | A04 | 430 | 47340 | 5.85 | D-284～L-326 | I |  |
| BrbZIP79 | Bra030310 | 456 | A04 | 151 | 17804.98 | 5.57 | L-13～M-59 | I |  |
| BrbZIP80 | Bra009288 | 444 | A10 | 147 | 17095.74 | 8.98 | K-53～Q-102 | I | At5g07160，AtbZIP72 |
| BrbZIP81 | Bra034913 | 669 | A08 | 222 | 25523.54 | 9.27 | K-98～Q-143 | I |  |
| BrbZIP82 | Bra009426 | 924 | A10 | 310 | 33889.16 | 7.68 | A-175～A-220 | I |  |
| BrbZIP83 | Bra027885 | 1122 | A09 | 373 | 42252.81 | 9.07 | T-232～N-279 | I |  |
| BrbZIP84 | Bra035464 | 531 | A01 | 176 | 20118.41 | 9.41 | T-37～N-84 | I |  |
| BrbZIP85 | Bra031173 | 1164 | A09 | 387 | 43383.91 | 9.03 | D-256～R-320 | I |  |
| BrbZIP86 | Bra034916 | 792 | A08 | 263 | 29001.47 | 9.22 | R-128～R-176 | I |  |
| BrbZIP87 | Bra032191 | 993 | A05 | 330 | 36368.39 | 8.14 | R-192～R-240 | I |  |
| BrbZIP88 | Bra030663 | 963 | A08 | 320 | 35361.26 | 5.34 | R-133～D-182 | I |  |
| BrbZIP89 | Bra031541 | 975 | A09 | 324 | 35840.8 | 5.77 | R-133～D-182 | I | At1g06850，AtbZIP52 |
| BrbZIP90 | Bra030637 | 1146 | A08 | 381 | 42568.36 | 6.14 | R-186～D-235 | I |  |
| BrbZIP91 | Bra015471 | 1179 | A10 | 392 | 43126.46 | 6.27 | R-187～D-236 | I |  |
| BrbZIP92 | Bra021735 | 1020 | A04 | 339 | 37841.27 | 6.1 | R-186～D-235 | I |  |
| BrbZIP93 | Bra018250 | 1125 | A05 | 374 | 41965.57 | 6.45 | R-183～D-232 | I |  |
| BrbZIP94 | Bra004582 | 1071 | A05 | 356 | 39658.68 | 6.22 | R-140～D-189 | I |  |
| BrbZIP95 | Bra016980 | 1029 | A04 | 342 | 38724.29 | 6.39 | R-133～D-182 | I |  |
| BrbZIP96 | Bra000195 | 1005 | A03 | 334 | 37381.16 | 6.23 | R-141～L-186 | I | At2g40620，AtbZIP18 |
| BrbZIP97 | Bra031172 | 1131 | A09 | 376 | 41955.99 | 9.44 | R-248～M-299 | I |  |
| BrbZIP98 | Bra030314 | 1383 | A04 | 460 | 49841.67 | 8.99 | R-324～D-373 | I |  |
| BrbZIP99 | Bra010722 | 1338 | A08 | 445 | 49390.57 | 6.45 | R-301～D-350 | I | At4g38900，AtbZIP29 |
| BrbZIP100 | Bra033582 | 1341 | A06 | 446 | 49360.86 | 7.55 | R-301～D-350 | I | At4g38900，AtbZIP29 |
| BrbZIP101 | Bra036142 | 2190 | A09 | 729 | 78641.41 | 8.39 | K-17～D-68 | S | At4g34590，AtbZIP11，ATB2 |
| BrbZIP102 | Bra010035 | 429 | A06 | 142 | 16065.22 | 8.98 | K-17～D-68 | S | At5g49450，AtbZIP1 |
| BrbZIP103 | Bra036025 | 573 | A09 | 190 | 22456.23 | 8.19 | R-75～Q-126 | S | At3g49760，AtbZIP5 |
| BrbZIP104 | Bra024424 | 750 | A06 | 249 | 28643.96 | 8.72 | S-148～Q-193 | S |  |
| BrbZIP105 | Bra031845 | 411 | A02 | 136 | 16114.44 | 9.88 | Q-22～Q-73 | S |  |
| BrbZIP106 | Bra027855 | 450 | A09 | 149 | 17075.46 | 8.62 | K-52～L-99 | S | At1g59530，AtbZIP4 |
| BrbZIP107 | Bra011780 | 741 | A01 | 246 | 28557.21 | 6.38 | K-139～V-185 | S |  |
| BrbZIP108 | Bra017850 | 723 | A03 | 240 | 27969.64 | 7.98 | K-128～L-173 | S |  |
| BrbZIP109 | Bra003500 | 405 | A07 | 134 | 15474.52 | 7.06 | K-22～Y-72 | S |  |
| BrbZIP110 | Bra007679 | 426 | A09 | 141 | 16183.32 | 6.69 | K-26～Y-76 | S | At3g62420，AtbZIP53 |
| BrbZIP111 | Bra024478 | 468 | A06 | 155 | 17277.27 | 5.72 | K-28～M-79 | S |  |
| BrbZIP112 | Bra037235 | 507 | A09 | 168 | 18495.56 | 4.96 | K-30～Y-80 | S |  |
| BrbZIP113 | Bra039631 | 507 | A07 | 168 | 18544.52 | 4.85 | K-30～M-81 | S | At2g18160，AtbZIP2，GBF5 |
| BrbZIP114 | Bra017664 | 462 | A03 | 153 | 17257.3 | 5.4 | K-28～Y-78 | S |  |
| BrbZIP115 | Bra011545 | 540 | A01 | 179 | 20119.9 | 6.14 | K-53～Y-103 | S |  |
| BrbZIP116 | Bra034639 | 429 | A08 | 142 | 16137.31 | 4.99 | K-17～Y-67 | S |  |
| BrbZIP117 | Bra015847 | 1077 | A07 | 358 | 35863.12 | 6.19 | K-38～Y-88 | S |  |
| BrbZIP118 | Bra008192 | 498 | A02 | 165 | 18042.14 | 6.61 | K-29～Y-79 | S |  |
| BrbZIP119 | Bra003755 | 540 | A07 | 179 | 19472.54 | 5.63 | K-39～Y-89 | S | At1g75390，AtbZIP44 |
| BrbZIP120 | Bra005971 | 483 | A03 | 160 | 19386.6 | 6.56 | R-62～R-114 | S | At5g08141，AtbZIP75 |
| BrbZIP121 | Bra035957 | 555 | A09 | 184 | 21515 | 7.27 | R-77～H-128 | S | At5g60830，AtbZIP70 |
| BrbZIP122 | Bra029353 | 315 | A02 | 104 | 12206.03 | 9.81 | M-1～H-48 | S |  |
| BrbZIP123 | Bra013005 | 549 | A03 | 182 | 21134.74 | 8.24 | R-72～H-123 | S |  |
| BrbZIP124 | Bra020735 | 516 | A02 | 171 | 19856.2 | 6.01 | K-80～H-130 | S |  |
| BrbZIP125 | Bra025418 | 519 | A06 | 172 | 20147.38 | 5.76 | K-81～H-131 | S | At5g38800，AtbZIP43 |
| BrbZIP126 | Bra008670 | 552 | A10 | 183 | 21118.33 | 8.94 | K-73～S-120 | S |  |
| BrbZIP127 | Bra006324 | 546 | A03 | 181 | 20807.95 | 6.23 | K-64～T-111 | S |  |
| BrbZIP128 | Bra023540 | 567 | A02 | 188 | 21920.16 | 6.22 | K-75～S-122 | S |  |
| BrbZIP129 | Bra038341 | 363 | A02 | 120 | 14130.87 | 9.13 | K-44～E-92 | S | At1g68880，AtbZIP8 |
| BrbZIP130 | Bra017359 | 525 | A09 | 174 | 20351.76 | 7.4 | K-85～S-132 | S |  |
| BrbZIP131 | Bra025144 | 513 | A06 | 170 | 19842.15 | 6.76 | K-88～E-136 | S |  |
| BrbZIP132 | Bra026523 | 270 | A02 | 89 | 10615.93 | 8.94 | M-1～E-45 | S |  |
| BrbZIP133 | Bra019715 | 582 | A06 | 193 | 22527.89 | 6.37 | K-89～Q-140 | S |  |
| BrbZIP134 | Bra026896 | 588 | A09 | 195 | 22657.76 | 5.45 | K-86～H-136 | S |  |
| BrbZIP135 | Bra026895 | 588 | A09 | 195 | 22657.76 | 5.45 | K-86～H-136 | S | At1g13600，AtbZIP58 |
| BrbZIP136 | Bra034925 | 720 | A08 | 233 | 25826.61 | 4.66 | D-149～K-168 |  | At1g42990，AtbZIP60 |
