# Supplementary material for: Genome-Wide Analysis of the bZIP Gene Family Identifies Two ABI5-Like bZIP Transcription Factors, BrABI5a and BrABI5b, as Positive Modulators of ABA Signalling in Chinese Cabbage
Source: PLoS One. 2016 Jul 14;11(7):e0158966. doi: 10.1371/journal.pone.0158966 (PMC4944949; doi:10.1371/journal.pone.0158966)
Supplement: S4 Table — (DOC) [file pone.0158966.s008.doc]

S4 Table. Sequence information of additional conserved motifs identified from 136 bZIP proteins in Chinese cabbage (*Brassica rapa*).

| S4 Table. Sequence information of additional conserved motifs identified from 136 bZIP proteins in Chinese cabbage (*Brassica rapa*). | | | | | |
| --- | --- | --- | --- | --- | --- |
| Motif No. | Length (aa) | E-Value | Multilevel Consensus Sequences | Number of BrabZIPs containing the MoNumber of BrabZIPs containing the Motif | Annotation |
| 1 | 29 | 2.3e-2084 | EKxDE[KR][RK]Q[KR]RM[IL][SK]NRESA[RA]RSR[AEL]RK[QK]AY[TLV] | 136 | bZIP super family |
| 2 | 94 | 6.9e-923 | [ML][AK]HY[EF]ELFR[MI]K[SA][AN]AAK[AN]DVF[HF]L[LIM]SGMW[KR]T[PS]AER[FC]FLW[IL]GGFR[PS]S[ED]LLK[VL]L[LA][PN][QH][FL][DE]P[LM]T[DE][QR]Q[VL][LM][GD][VI][CN][NS]L[QR]Q[ST][SC]QQAE[DE]ALSQG[ML]E[KS]LQ[QH][ST]LA[DE]T[LV] | 16 |  |
| 3 | 21 | 3.9e-587 | ELEx[QKE]VxQL[QR]EENxTLSx[KQ]Lx | 122 |  |
| 4 | 57 | 3.6e-479 | [YG][MI][GP]QMA[MS]A[ML][GE][KRNQ]L[GE][AT]L[EV][GS]F[IV][RN]QAD[HN]LR[LHQ][QE]TLQQ[ML][HIR][RK][IV]LTTRQ[AS]AR[GA][LF]L[AV][IL][GH][ED]Y[FS][QSH]RLR | 15 |  |
| 5 | 57 | 8.2e-392 | I[DS][ST][SN]G[LD][QG][AFP][HS]ST[MGI][GN][NTS]G[IA][AL]AF[ED][MA]E[YH][GS][RH]W[LI]EE[QK]N[RK][QR][IM][SNC]E[LI]R[TS][AV]L[QN][AG][HQ][VA][SG]DVELRL[LI]V[DE][GN][VA] | 17 | DOG1 superfamily |
| 6 | 34 | 6.0e-384 | RQGSLTLPR[TDP][LI][SC][KQ]KTV[DE]EVWK[DE][IL]QQ[DKG][DKG][GN][GS]G[GSN][GS][GS][GS] | 25 |  |
| 7 | 29 | 1.4e-328 | Q[RK]Q[QP]TLGE[MI]TLE[DE][FL]L[LV][KR]AGVV[RT]E[TD]xTGSN | 27 |  |
|  |  |  |  |  |  |
| 8 | 47 | 2.70E-304 | [LF]QRDT[NM]GL[TS][NVS][EQ]N[NT]ELK[LFM]RLQ[AST]MEQQ[AV][HQK]L[RQ][DN]AL[NS][ED][AK]L[KR][EKG]E[VL][QE][RH][LM]K[FLMV][AL][TIA] | 15 |  |
| 9 | 28 | 3.90E-286 | G[SGE][SP]GKD[FL]GSMN[ML]DE[LF]LK[SN][IV]W[TS]AEE[NT][QN]AM | 22 |  |
| 10 | 41 | 2.30E-235 | [DV][TAM][TN]Q[KHL][YF][DV][TAK][AIM][EA][AV][ED]N[RS][IV]L[RK]A[QD][IL]E[ET]L[RS][TH][RK][LV][KE][SM][LA][NE][ED][IT][VI][KDE][RFL][VL][TE]GT[NS] | 20 |  |
| 11 | 21 | 3.40E-183 | L[EK]E[ES]R[LS][KR]L[RK]QL[EL]QEL[QK]RARQQ | 37 |  |
| 12 | 15 | 2.60E-150 | [GAT]RQ[SNG]S[LI]YSLT[LF]DE[FVL]Q | 24 |  |
| 13 | 57 | 4.40E-132 | [QG][VAL][AG]PW[VI]QYHQLPSM[PLT]Q[PG][QR][SA]F[MF]PY[PL]V[AS]DMQ[AV]MVSQ[SAT]SLMGGL[SY]DTQTPGRKRVASGEV | 4 |  |
| 14 | 15 | 5.10E-82 | [GP][DS][PK][KR][RQ][CKQ]LRRT[SL][ST][AG][PS][WF] | 18 |  |
| 15 | 21 | 1.10E-69 | [HA][PF][VMP]PP[HR][PRG][VY][IV][AI][YGS][SE]P[LSQ][PH][HQ][PF][YG]MWG | 14 |  |
| 16 | 11 | 1.00E-65 | ALSS[LS]W[LA][AT]RPR | 16 |  |
| 17 | 49 | 3.30E-63 | [SF]SDANR[FL]SHD[IM]S[RL]M[LPS][DE][HN]PPK[KN][IL]GHRRAHSEILTLPDDLSFD[SF]DLGVVG[AG] | 4 |  |
| 18 | 15 | 1.40E-55 | [PRS]R[GP]RH[RQ][RH]S[VI]S[VMD][DS][GSI][AGS][FS] | 20 |  |
| 19 | 168 | 2.10E-54 | GSPDGKRLH[QR]WFHEGGAGTLMDYSMCTEVFQFDISPGAIVPSSVSNITREHLQN[GV]TTTRDKRMKNRRILEGL[PR]VSRLASELNITEAQASKDAQNK[ST]FHGKANTKPTSSSMVVSVLLDPREVVD[PS]ETDRVMPSNPKSLSR[IV]FVVVLLDSVKYVTYSCVLPRSGLHLVAT | 2 |  |
|  |  |  |  |  |  |
| 20 | 21 | 4.30E-51 | [TG][PT]P[HV]PY[VP][AT]MYPPG[GW][MV]YA[HP]P[SG]M | 7 |  |
| 21 | 41 | 1.30E-56 | L[FL]SS[SL]PL[PA]PPATVLSLNS[GH][AS][GI]F[ET][FH][LF]D[NE][QS][DA]P[LF][VG][TC][SFP][NG][KS][NK][LRS][HG]SH | 5 |  |
| 22 | 158 | 3.70E-46 | [IV]PKLPLPSCSRPKKGEG[KR]SKLKK[AV]AS[IV]SFIGI[LM]FFMFLFG[MT]LVPFMNVDNGGD[RS]GLAKYEGRR[HY]YDEHRGKVLMVGDGSDVRRESVCS[GI]RDSCGGVEGRLSNASEPLFASLYVPRNDGLVKIDGNLIIHSVLA[NS]EKAKKN[IV]SETIKS[EK]E[AP][DE]LTVPG[AV]P | 2 |  |
| 23 | 21 | 1.20E-43 | [NP][PN]NSIFQDFLN[GKR]PLNQEP[TP][PI]P | 5 |  |
| 24 | 29 | 7.40E-49 | FL[DN][HQ][QR][RN]L[LI]L[NS][VL][DE]N[SK]A[LI]KQR[IL][AE][AS][LI]AQ[DE]K[IL][FI] | 7 |  |
| 25 | 21 | 1.00E-46 | [DF][DS][LD][EDG]S[DEM][DE]DL[FL][SC][AMS]Y[ML][DN][LMV][DE][KN][LFI][DNG][SA] | 13 |  |
